# Supplementary material for: Effects of a community-driven water, sanitation, and hygiene intervention on diarrhea, child growth, and local institutions: A cluster-randomized controlled trial in rural Democratic Republic of Congo
Source: PLoS Med. 2025 Mar 6;22(3):e1004524. doi: 10.1371/journal.pmed.1004524 (PMC11884671; doi:10.1371/journal.pmed.1004524)
Supplement: S1 Table — (DOCX) [file pmed.1004524.s001.docx]

**S1 Table. Randomization strata**

| Province | Stratum | Villages-per-cluster | Total villages in stratum | Intervention villages in stratum | Control villages in stratum | Total clusters in stratum | Intervention clusters in stratum | Control clusters in stratum |
| --- | --- | --- | --- | --- | --- | --- | --- | --- |
| Kongo Central | 1 | 1-2 | 15 | 8 | 7 | 12 | 6 | 6 |
|  | 2 | 3-4 | 13 | 6 | 7 | 4 | 2 | 2 |
|  | 3 | 5,7 | 12 | 7 | 5 | 2 | 1 | 1 |
| Kasai | 1 | 1-2 | 37 | 19 | 18 | 26 | 13 | 13 |
|  | 2 | 3-5 | 39 | 21 | 18 | 11 | 6 | 5 |
|  | 3 | 10,12 | 22 | 12 | 10 | 2 | 1 | 1 |
| Kasai Central | 1 | 1-2 | 34 | 8 | 26 | 25 | 6 | 19 |
|  | 2 | 3-4 | 34 | 8 | 26 | 10 | 2 | 8 |
|  | 3 | 6-7 | 13 | 0 | 13 | 2 | 0 | 2 |
| South Kivu | 1 | 1-2 | 20 | 9 | 11 | 13 | 6 | 7 |
|  | 2 | 4-8,10 | 74 | 39 | 35 | 12 | 6 | 6 |
|  | 3 | 12,14 | 26 | 12 | 14 | 2 | 1 | 1 |
| Total |  |  | 339 | 149 | 190 | 121 | 50 | 71 |
