# Supplementary material for: Effects of a community-driven water, sanitation, and hygiene intervention on diarrhea, child growth, and local institutions: A cluster-randomized controlled trial in rural Democratic Republic of Congo
Source: PLoS Med. 2025 Mar 6;22(3):e1004524. doi: 10.1371/journal.pmed.1004524 (PMC11884671; doi:10.1371/journal.pmed.1004524)
Supplement: S2 Table — (DOCX) [file pmed.1004524.s002.docx]

**S2 Table. Intervention effects on WASH institutions index and index sub-components**

|  | Control | | | Intervention | | |  | CI 95% | |
| --- | --- | --- | --- | --- | --- | --- | --- | --- | --- |
| Outcomes | n | Mean | SD | n | Mean | SD | ITT | Lower Bound | Upper Bound |
| WASH institutions index | 185 | 0.00 | 1.00 | 144 | 0.46 | 0.75 | 0.40 | 0.16 | 0.65 |
| Committee (y/n) | 185 | 0.70 | 0.46 | 144 | 0.97 | 0.16 | 0.21 | 0.10 | 0.32 |
| Committee mtg freq* | 88 | 2.91 | 1.49 | 104 | 2.62 | 1.61 | -0.29 | -0.79 | 0.20 |
| WASH expenditures (CDF per month, IHS) | 130 | 2.12 | 4.07 | 140 | 3.28 | 4.58 | 1.14 | 0.07 | 2.20 |
| Track health (y/n) | 185 | 0.69 | 0.46 | 144 | 0.84 | 0.37 | 0.16 | 0.07 | 0.25 |
| Track sanitation (y/n) | 185 | 0.74 | 0.44 | 144 | 0.78 | 0.41 | 0.05 | -0.06 | 0.15 |

ITT = intention-to-treat effect estimate. CDF = Congolese francs. IHS = inverse hyperbolic spline. Effects are estimated with models that include controls for randomisation blocks based on province and number of villages per cluster. There were 121 clusters in total. The WASH institutions index was calculated by rescaling each variable in the index (eg,presence of WASH committee) so that higher values imply better outcomes, then standardising relative to the control group, following Kling *et al*. Effects are in standard deviation units. The index values range from -2.35 to 2.12.
*Committee meeting frequency is coded 1-6, where 1=weekly, 2=Fortnightly, 3=Monthly, 4=Every 3 months, 5=Every 6 or more months, 6=No regular schedule, based on needs
