# Supplementary material for: Effects of a community-driven water, sanitation, and hygiene intervention on diarrhea, child growth, and local institutions: A cluster-randomized controlled trial in rural Democratic Republic of Congo
Source: PLoS Med. 2025 Mar 6;22(3):e1004524. doi: 10.1371/journal.pmed.1004524 (PMC11884671; doi:10.1371/journal.pmed.1004524)
Supplement: S3 Table — (DOCX) [file pmed.1004524.s003.docx]

**S3 Table. Intervention effects on all secondary outcomes, including index sub-components**

|  | Control | | | Intervention | | |  | CI 95% | |
| --- | --- | --- | --- | --- | --- | --- | --- | --- | --- |
| Outcomes | n | Prevalence/  Mean | SD | N | Prevalence/  Mean | SD | ITT | Lower | Upper |
| Improved water source | 1845 | 43% |  | 1437 | 74% |  | 0.24 | 0.12 | 0.36 |
| Improved sanitation | 1843 | 14% |  | 1437 | 34% |  | 0.18 | 0.10 | 0.25 |
| Water governance perception index | 1845 | 0.00 | 1.00 | 1438 | 0.22 | 0.88 | 0.19 | 0.04 | 0.34 |
| Committee selected fairly | 600 | 1.76 | 0.81 | 1119 | 1.82 | 0.76 | 0.06 | -0.04 | 0.17 |
| Committee treats community fairly | 621 | 4.15 | 1.31 | 1158 | 4.07 | 1.31 | -0.09 | -0.27 | 0.09 |
| Committee manages money well | 635 | 3.25 | 1.12 | 721 | 3.31 | 1.14 | 0.05 | -0.13 | 0.23 |
| Comm responds breakdown well | 972 | 3.69 | 1.18 | 1227 | 3.67 | 1.23 | 0.01 | -0.13 | 0.15 |
| Confidence committee will solve reported issue | 1009 | 3.10 | 1.05 | 1227 | 3.12 | 1.03 | 0.02 | -0.13 | 0.17 |
| Satisfaction with water access (1-5) | 1845 | 2.76 | 1.49 | 1438 | 3.38 | 1.43 | 0.56 | 0.27 | 0.85 |
|  |  |  |  |  |  |  |  |  |  |
| Time spent collecting water (IHS) | 1845 | 4.17 | 2.07 | 1438 | 4.04 | 2.05 | 0.03 | -0.20 | 0.27 |
| HHs pay for water (Dummy) | 1845 | 10% |  | 1438 | 23% |  | 0.09 | 0.00 | 0.19 |
| Water use expenditure (weekly) | 137 | 153 | 217 | 303 | 254 | 351 | 114 | -16 | 243 |
| Hygiene and behaviour index | 1845 | 0.00 | 1.00 | 1438 | 0.30 | 1.07 | 0.23 | 0.12 | 0.34 |
| Handwashing score (0-10) | 1845 | 2.79 | 2.22 | 1438 | 3.41 | 2.16 | 0.43 | 0.19 | 0.67 |
| Open defecation (%) | 1843 | 32% |  | 1437 | 27% |  | -0.03 | -0.08 | 0.01 |
| Self-reported handwashing with soap/ash (%) | 1843 | 67% |  | 1437 | 82% |  | 0.09 | 0.06 | 0.13 |
| Frequency of latrine cleaning over past 2 weeks | 761 | 4.89 | 4.26 | 648 | 5.00 | 4.06 | 0.25 | -0.32 | 0.82 |
| Water in pot is clean and covered (%) | 1669 | 57% |  | 1273 | 57% |  | 0.02 | -0.03 | 0.07 |
| Water treated for consumption, any method (%) | 1669 | 3% |  | 1273 | 7% |  | 0.03 | 0.01 | 0.05 |
|  |  |  |  |  |  |  |  |  |  |
| Handwashing action | 616 | 15% |  | 489 | 17% |  | 0.00 | -0.04 | 0.03 |
| Handwashing with soap/ash | 616 | 2% |  | 489 | 4% |  | 0.01 | -0.01 | 0.03 |
| Water point has water | 380 | 97% |  | 266 | 85% |  | -0.11 | -0.18 | -0.05 |
| WP FIB MPN/100mL | 366 | 79.31 | 37.92 | 221 | 72.41 | 42.56 | -9.46 | -18.85 | -0.06 |
| WP FIB log10 MPN/100mL | 366 | 1.71 | 0.60 | 221 | 1.58 | 0.70 | -0.17 | -0.32 | -0.02 |
| HH FIB MPN/100mL | 1098 | 82.27 | 35.05 | 851 | 79.44 | 37.71 | -3.87 | -9.59 | 1.86 |
| HH FIB log10 MPN/100mL | 1098 | 1.76 | 0.53 | 851 | 1.72 | 0.58 | -0.06 | -0.15 | 0.04 |
|  |  |  |  |  |  |  |  |  |  |
| Weight for age | 1223 | -1.17 | 1.16 | 919 | -1.14 | 1.16 | 0.06 | -0.04 | 0.15 |
| Weight for length | 1224 | 0.14 | 1.07 | 918 | 0.22 | 1.03 | 0.10 | -0.02 | 0.22 |
| Life satisfaction & self-esteem index | 1843 | 0.00 | 1.00 | 1435 | 0.03 | 0.98 | 0.01 | -0.12 | 0.14 |
| Life satisfaction (WVS) | 1843 | 4.87 | 3.20 | 1435 | 5.04 | 2.89 | 0.20 | -0.10 | 0.49 |
| Feel I am person of worth (Rosenberg) | 1843 | 2.25 | 0.90 | 1435 | 2.19 | 0.90 | -0.03 | -0.13 | 0.07 |
| Feel that I have good qualities (Rosenberg) | 1843 | 2.42 | 0.74 | 1435 | 2.36 | 0.75 | -0.02 | -0.10 | 0.05 |
| Inclined to feel I am a failure (Rosenberg) | 1843 | 1.43 | 1.11 | 1435 | 1.49 | 1.05 | -0.04 | -0.16 | 0.08 |
| Able to do things as well as other people (Rosenberg) | 1843 | 2.44 | 0.75 | 1435 | 2.40 | 0.76 | 0.01 | -0.06 | 0.08 |
| Feel have not much to be proud of (Rosenberg) | 1843 | 0.98 | 0.98 | 1435 | 0.99 | 0.92 | -0.05 | -0.14 | 0.04 |
| Take a positive attitude towards self (Rosenberg) | 1843 | 2.35 | 0.78 | 1435 | 2.37 | 0.74 | 0.08 | -0.01 | 0.17 |
| I am satisfied with myself (Rosenberg) | 1843 | 2.03 | 1.02 | 1435 | 2.12 | 0.96 | 0.16 | 0.07 | 0.26 |
| Wish could have more respect for myself (Rosenberg) | 1843 | 0.42 | 0.60 | 1435 | 0.40 | 0.57 | -0.07 | -0.13 | 0.00 |
| Certainly feel useless at times (Rosenberg) | 1843 | 1.33 | 1.10 | 1435 | 1.40 | 1.07 | -0.03 | -0.16 | 0.10 |
| At times think I am no good at all (Rosenberg) | 1843 | 1.32 | 1.09 | 1435 | 1.43 | 1.07 | 0.00 | -0.12 | 0.12 |
|  |  |  |  |  |  |  |  |  |  |
| Psychological well-being index | 1843 | 0.00 | 1.00 | 1435 | 0.05 | 0.99 | 0.06 | -0.06 | 0.17 |
| Felt cheerful last 2 weeks (WHO) | 1843 | 2.59 | 1.72 | 1435 | 2.64 | 1.64 | 0.05 | -0.12 | 0.22 |
| Felt calm & relaxed last 2 weeks (WHO) | 1843 | 2.62 | 1.68 | 1435 | 2.68 | 1.59 | 0.06 | -0.11 | 0.22 |
| Felt active & vigorous last 2 weeks (WHO) | 1843 | 2.57 | 1.64 | 1435 | 2.61 | 1.54 | 0.04 | -0.13 | 0.21 |
| Woke up fresh & rested last 2 weeks (WHO) | 1843 | 2.58 | 1.62 | 1435 | 2.56 | 1.53 | 0.02 | -0.14 | 0.18 |
| Daily life filled with things that interest last 2 weeks (WHO) | 1843 | 2.25 | 1.76 | 1435 | 2.30 | 1.70 | 0.12 | -0.06 | 0.29 |
| Felt unable to control important things last month (Cohen) | 1843 | 2.95 | 1.26 | 1435 | 2.92 | 1.12 | -0.06 | -0.17 | 0.05 |
| Felt confident about ability to handle personal problems last month (Cohen) | 1843 | 3.09 | 1.27 | 1435 | 3.05 | 1.17 | 0.02 | -0.08 | 0.13 |
| Felt confident things were going your way last month (Cohen) | 1843 | 2.81 | 1.33 | 1435 | 2.89 | 1.25 | 0.10 | -0.02 | 0.23 |
| Felt difficulties were piling up could not overcome them last month (Cohen) | 1843 | 2.39 | 1.28 | 1435 | 2.56 | 1.20 | 0.10 | -0.03 | 0.23 |
|  |  |  |  |  |  |  |  |  |  |
| School attendance (days past week) | 299 | 3.21 | 2.72 | 226 | 2.74 | 2.72 | -0.38 | -0.82 | 0.07 |

ITT = intention-to-treat effect estimate; HH = household; FIB = fecal indicator bacteria; MPN = most probable number; IHS = inverse hyperbolic spline; WHO = World Health Organization; WVS = World Values Survey. Effects are estimated with models that include controls for randomisation blocks based on province and number of villages per cluster. There were 121 clusters in total. The WASH governance perceptions index and the hygiene and behavior index were calculated by rescaling each variable in the index (eg, satisfaction with water access) so that higher values imply better outcomes, then standardising relative to the control group, following Kling *et al*. Effects are in standard deviation units.
