# Supplementary material for: Effects of a community-driven water, sanitation, and hygiene intervention on diarrhea, child growth, and local institutions: A cluster-randomized controlled trial in rural Democratic Republic of Congo
Source: PLoS Med. 2025 Mar 6;22(3):e1004524. doi: 10.1371/journal.pmed.1004524 (PMC11884671; doi:10.1371/journal.pmed.1004524)
Supplement: S4 Table — (DOCX) [file pmed.1004524.s004.docx]

**S4 Table. Intervention effects on all primary outcomes, separately by province (pre-specified)**

|  |  | Control | | | Intervention | | |  | CI 95% | |
| --- | --- | --- | --- | --- | --- | --- | --- | --- | --- | --- |
| Outcomes | Province | n | Prevalence/Mean | SD | n | Prevalence/Mean | SD | ITT | Lower Bound | Upper Bound |
| Diarrhea prevalence | Kongo Central | 146 | 17% |  | 163 | 10% |  | -0.07 | -0.15 | 0.00 |
|  | Kasai | 599 | 40% |  | 679 | 40% |  | 0.00 | -0.09 | 0.09 |
|  | Kasai Central | 781 | 49% |  | 196 | 59% |  | 0.10 | 0.00 | 0.19 |
|  | South Kivu | 784 | 41% |  | 724 | 37% |  | -0.05 | -0.10 | 0.01 |
|  |  |  |  |  |  |  |  |  |  |  |
| Length-for-age Z-score | Kongo Central | 96 | -1.59 | 1.32 | 93 | -1.75 | 1.67 | -0.18 | -0.47 | 0.11 |
|  | Kasai | 319 | -2.05 | 1.60 | 328 | -1.86 | 1.60 | 0.20 | -0.05 | 0.44 |
|  | Kasai Central | 409 | -2.12 | 1.71 | 103 | -2.58 | 1.49 | -0.42 | -0.80 | -0.04 |
|  | South Kivu | 399 | -2.49 | 1.48 | 395 | -2.50 | 1.50 | 0.02 | -0.16 | 0.19 |
|  |  |  |  |  |  |  |  |  |  |  |
| WASH institutions index | Kongo Central | 18 | 0.27 | 0.66 | 20 | 0.59 | 0.57 | 0.31 | -0.06 | 0.68 |
|  | Kasai | 43 | -0.48 | 1.10 | 48 | 0.21 | 0.75 | 0.70 | 0.21 | 1.19 |
|  | Kasai Central | 65 | -0.18 | 0.85 | 16 | 0.28 | 0.61 | 0.35 | 0.03 | 0.68 |
|  | South Kivu | 59 | 0.46 | 0.96 | 60 | 0.67 | 0.78 | 0.23 | -0.25 | 0.71 |

ITT = intention-to-treat effect estimate. Effects are estimated with models that include controls for randomisation blocks based on province and number of villages per cluster. There were 121 clusters in total. The WASH institutions index was calculated by rescaling each variable in the index (eg,presence of WASH committee) so that higher values imply better outcomes, then standardising relative to the control group, following Kling *et al*. Effects are in standard deviation units.
