# Supplementary material for: Effects of a community-driven water, sanitation, and hygiene intervention on diarrhea, child growth, and local institutions: A cluster-randomized controlled trial in rural Democratic Republic of Congo
Source: PLoS Med. 2025 Mar 6;22(3):e1004524. doi: 10.1371/journal.pmed.1004524 (PMC11884671; doi:10.1371/journal.pmed.1004524)
Supplement: S7 Table — (DOCX) [file pmed.1004524.s007.docx]

**S7 Table. Intervention effects on diarrhea and length-for-age z score, sex-by-intervention interaction models**

|  | (1) | (2) | (3) | (4) |
| --- | --- | --- | --- | --- |
|  | Diarrhea - Simple | Diarrhea - Interaction | Height for Age - Simple | Height for Age - Interaction |
| Treatment | -0.010 | -0.004 | -0.014 | 0.071 |
|  | (0.022) | (0.027) | (0.070) | (0.098) |
|  |  |  |  |  |
| Child's sex | 0.010 | 0.014 | 0.134** | 0.206** |
|  | (0.014) | (0.018) | (0.062) | (0.084) |
|  |  |  |  |  |
| Child's age (years) | -0.043*** | -0.043*** | -0.233*** | -0.233*** |
|  | (0.004) | (0.004) | (0.033) | (0.033) |
|  |  |  |  |  |
| Kasai Province # cluster_strata=2 | 0.267*** | 0.267*** | -0.085 | -0.089 |
|  | (0.050) | (0.050) | (0.200) | (0.199) |
|  |  |  |  |  |
| Kasai Province # cluster_strata=3 | 0.232*** | 0.232*** | 0.222 | 0.213 |
|  | (0.078) | (0.078) | (0.172) | (0.174) |
|  |  |  |  |  |
| Kasai Central Province # cluster_strata=2 | 0.372*** | 0.372*** | -0.347 | -0.351 |
|  | (0.059) | (0.058) | (0.261) | (0.260) |
|  |  |  |  |  |
| Kasai Central Province # cluster_strata=3 | 0.335*** | 0.335*** | -0.143 | -0.150 |
|  | (0.063) | (0.063) | (0.168) | (0.167) |
|  |  |  |  |  |
| Sud-Kivu Province # cluster_strata=2 | 0.273*** | 0.273*** | -0.729*** | -0.737*** |
|  | (0.041) | (0.041) | (0.161) | (0.159) |
|  |  |  |  |  |
| Sud-Kivu Province # cluster_strata=3 | 0.268*** | 0.268*** | -0.240 | -0.242 |
|  | (0.042) | (0.042) | (0.164) | (0.163) |
|  |  |  |  |  |
| Treatment # Female |  | -0.011 |  | -0.168 |
|  |  | (0.028) |  | (0.122) |
|  |  |  |  |  |
| Constant | 0.235*** | 0.233*** | -1.323*** | -1.354*** |
|  | (0.039) | (0.040) | (0.182) | (0.185) |
| Observations | 4072 | 4072 | 2142 | 2142 |
| Wald_F |  | 0.159 |  | 1.883 |
| Wald_p |  | 0.691 |  | 0.173 |

Coefficients and standard errors from linear models of the primary outcomes on intervention group, fixed effects for randomization strata, fixed effects for child sex, and intervention-by-sex interaction terms. Standard errors are clustered by cluster (group of villages). There were 121 clusters in total. The WASH institutions index was calculated by rescaling each variable in the index (eg,presence of WASH committee) so that higher values imply better outcomes, then standardising relative to the control group, following Kling *et al*. Effects are in standard deviation units. The Wald F is a test statistic for the null hypothesis that all of the interaction term coefficients are zero. * p<.1, ** p<.05, *** p<.01"
