# Supplementary material for: Effects of a community-driven water, sanitation, and hygiene intervention on diarrhea, child growth, and local institutions: A cluster-randomized controlled trial in rural Democratic Republic of Congo
Source: PLoS Med. 2025 Mar 6;22(3):e1004524. doi: 10.1371/journal.pmed.1004524 (PMC11884671; doi:10.1371/journal.pmed.1004524)
Supplement: S8 Table — (DOCX) [file pmed.1004524.s008.docx]

**S8 Table. Variable definitions**

**Descriptions of primary and secondary outcomes, including index subcomponents**

| **Category** | **Outcomes** | **Definition** |
| --- | --- | --- |
|  |  |  |
| **Primary Outcome** | **Diarrhea** | Prevalence of diarrhea in last 7 days of children under 5 (caregiver reported):  1. Yes to ‘diarrhea’ , or  2. Yes to ‘three or more stools” AND ‘watery or soft stool’, or  3. Yes to blood in the stool |
|  |  |  |
| **Primary Outcome** | **Length for age** | Length for age z score for children under 5.  The sample does not include observations with implausible z-scores. |
|  |  |  |
| **Primary Outcome** | **WASH institutions index** | The index is built from: (1) Presence of water committee; (2) Frequency of meeting; (3) Average amount spent per month for water activities excluding maintenance (inverse hyperbolic sine); (4) Tracks health conditions; (5) Tracks hygiene and sanitation |
| Subcomponent | Presence of water committee | 1 if the village has a water committee  0 Otherwise |
| Subcomponent | Frequency of meeting | Frequency of committee meeting. The variable ranges from 1 to 6:  1 Weekly  2 Fortnightly  3 Monthly  4 Every 3 months  5 Every 6 or more months  6 No regular schedule, based on needs |
| Subcomponent | Average amount spent per month for water activities excluding maintenance. | Average amount spent per month for water activities excluding maintenance. An inverse hyperbolic sine transformation has been applied to the variable. |
| Subcomponent | Tracks health conditions | 1 if village keeps track of community health conditions  0 Otherwise |
| Subcomponent | Tracks hygiene and sanitation | 1 if village keeps track of WASH practices  0 Otherwise |
|  |  |  |
| **Secondary Outcome** | **Water governance perception index** | Water governance perception index is constructed based on the following variables: fairness of selection of water governance entity; perception of fair treatment; confidence in managing money of committee; confidence in committee response to breakdowns; confidence in committee management; Satisfaction with water access |
| Subcomponent | Committee selected fairly | The extent to which the process of choosing committee members was fair  The answer choices are:  Not fair at all;  Somewhat fair;  Fair;  Fully fair |
| Subcomponent | Committee treats community fairly | The extent to which the water committee treats you fairly  The answer choices are:  Very unfair  Somewhat unfair  Neither fair nor unfair  Somewhat fair  Very fair |
| Subcomponent | Committee manages money well | How well the water committee manages money  The answer choices are the following:  Very badly  Badly  Neither bad nor good  Good  Very good |
| Subcomponent | Comm responds breakdown well | How well the water committee responds to breakdowns  The answer choices are the following:  Very badly  Badly  Neither bad nor good  Good  Very good |
| Subcomponent | Confidence committee will solve reported issue | Level of confidence that water committee will solve issue respondent brings up  The answer choices are the following:  Not confident at all  Not very confident  Somewhat confident  Very confident |
| Subcomponent | Satisfaction with water access (1-5) | Level of satisfaction of respondent with your access to water  The answer choices are the following:  Not satisfied at all  Not satisfied  Indifferent  Satisfied  Very satisfied |
|  |  |  |
| **Secondary Outcome** | **Improved water source** | 1 if Household’s Primary source of drinking water is improved source (JMP definition) and 0 otherwise.   - Improved main drinking source includes Piped into dwelling, Piped into plot, Piped/public tap, Tube well or borehole, Protected dug well, Protected spring, Rainwater, Tanker truck. - Unimproved main drinking source includes Unprotected dug well, Unprotected spring, Surface water, No other main source |
|  |  |  |
| **Secondary Outcome** | **Time spent collecting water (IHS)** | Each household was asked to list household members who participated on previous day, and the number of trips undertaken by the household. For each trip, the collector was identified and the time spent for the round trip was collected. For each household, we sum time spent for each trip in minutes to get the total time spent collecting water per household. To estimate treatment effects, an inverse hyperbolic sine transformation was applied due to the large number of zeroes. |
|  |  |  |
| **Secondary Outcome** | **HHs pay for water (Dummy)** | 1 if household pay for water  0 Otherwise |
|  |  |  |
| **Secondary Outcome** | **Water use expenditure (weekly)** | Estimated household’s weekly expenditure for water use (in CDF, Congolese Franc) |
|  |  |  |
| **Secondary Outcome** | **Improved sanitation** | Household uses an improved latrine (JMP definition)   - Improved sanitation includes options such as Flush / Pour Flush: to Piped Sewer System, Flush / Pour Flush: to Septic Tank, Flush / Pour Flush: to Pit Latrine, Ventilated Improved Pit Latrine (VIP), Pit Latrine with Slab, Composting Latrine. - Unimproved sanitation includes Flush / Pour Flush: to Elsewhere, Flush / Pour Flush: to Don’t Know Where, Pit Latrine without Slab / Open Pit, Bucket Latrine, Hanging Toilet, No Facilities (Bush, Open Field, River), Other (specify) |
|  |  |  |
| **Secondary Outcome** | **Hygiene and behaviour index** | Self-reports of: Knowledge: Caregiver knows how and when to wash hands; what causes diarrhea. Sanitation practices: cleanliness of household area and latrine (presence of flies and fecal matter); open defecation; observed indicators of toilet use –worn pathway, presence of water; improvements to latrine; disposes of child feces safely (JMP definition). Water storage practices: has a clean pot for water that is covered. |
| Subcomponent | Handwashing score (0-10) | Self-report of: Knowledge: Caregiver knows how and when to wash hands. The variables considered are the following: count 1 if respondent mentioned unprompted that they wash hands with soap/ash in critical juncture X (= 0 if they say no or they say it prompted). The counts are then added up to create a score.  The considered junctures are the following: After toilet; after washing baby’s bottom/changing; after eating; before preparing food; before eating; before feeding/breastfeeding baby; before or after handling children; after taking care of pets or farm animals; after coughing/sneezing; after coming back from the fields. |
| Subcomponent | Open defecation (%) | 1 if no defecation facility used and 0 otherwise |
| Subcomponent | Self-reported handwashing with soap/ash (%) | 1 if respondent washed their hands with soap/ash at least once since previous day  0 Otherwise |
| Subcomponent | Frequency of latrine cleaning over past 2 weeks | Number of times the latrine has been cleaned in the past 2 weeks |
| Subcomponent | Water pot is clean and covered (%) | 1 if the pot has clean water and is covered  0 Otherwise.  This indicator is measured via actual observations |
| Subcomponent | Water treated for consumption, any method (%) | 1 if drinking water stored in household is treated with any product/method for safe consumption  0 otherwise |
|  |  |  |
| **Secondary Outcome** | **Life satisfaction & self-esteem index** | Summary index of 11 questions  The 11 questions include the (1) life satisfaction question as defined by the World Values Survey and (2) the 10 questions as defined in Rosenberg's Self-Esteem Scale. |
| Subcomponent | Life satisfaction (WVS) | All things considered, on a scale of 1 to 10, how satisfied are you with your life as a whole?  1 means completely dissatisfied  10 means completely satisfied |
| Subcomponent | Feel I am person of worth (Rosenberg) | I feel I am a person of worth, at least on an equal plane with others.  Tell me to what extent you: Strongly agree, Agree, Disagree, or Strongly disagree with this statement about you. |
| Subcomponent | Feel that I have good qualities (Rosenberg) | I feel that I have a number of good qualities.  Tell me to what extent you: Strongly agree, Agree, Disagree, or Strongly disagree with this statement about you. |
| Subcomponent | Inclined to feel I am a failure (Rosenberg) | All in all, I am inclined to feel that I am a failure.  Tell me to what extent you: Strongly agree, Agree, Disagree, or Strongly disagree with this statement about you. |
| Subcomponent | Able to do things as well as oth people (Rosenberg) | I am able to do things as well as most other people.  Tell me to what extent you: Strongly agree, Agree, Disagree, or Strongly disagree with this statement about you. |
| Subcomponent | Feel have not much to be proud of (Rosenberg) | I feel I do not have much to be proud of.  Tell me to what extent you: Strongly agree, Agree, Disagree, or Strongly disagree with this statement about you. |
| Subcomponent | Take a positive attitude towards self (Rosenberg) | I take a positive attitude toward myself.  Tell me to what extent you: Strongly agree, Agree, Disagree, or Strongly disagree with this statement about you. |
| Subcomponent | I am satisfied with myself (Rosenberg) | On the whole, I am satisfied with myself.  Tell me to what extent you: Strongly agree, Agree, Disagree, or Strongly disagree with this statement about you. |
| Subcomponent | Wish could have more respect for myself (Rosenberg) | I wish I could have more respect for myself.  Tell me to what extent you: Strongly agree, Agree, Disagree, or Strongly disagree with this statement about you. |
| Subcomponent | Certainly feel useless at times (Rosenberg) | I certainly feel useless at times.  Tell me to what extent you: Strongly agree, Agree, Disagree, or Strongly disagree with this statement about you. |
| Subcomponent | At times think I am no good at all (Rosenberg) | At times I think I am no good at all.  To what extent do respondents: Strongly agree, Agree, Disagree, or Strongly disagree with this statement about them |
|  |  |  |
| **Secondary Outcome** | **Psychological well-being index** | Summary index of 9 questions on well-being in last 2 weeks and stress in last 4 weeks.  The questions used here belong to two different sets of questions. The first set is the 5 WHO questions on well-being, and the second set is the Cohen stress scale 4 questions.  Each of the five WHO statements on well-being refers to how respondents have been feeling over the last two weeks. The answer choices are the following: At no time; Some of the time; Less than half of the time; More than half of the time; Most of the time; All of the time.  Questions in the Cohen stress scale, ask the respondent about their feelings and thoughts during THE LAST MONTH. The answer choices are the following: Never; Almost never; Sometimes; Fairly often; Very often. |
| Subcomponent | Felt cheerful last 2 weeks (WHO) | Over the last two weeks, I have felt cheerful and in good spirits.  The answer choices are the following: At no time; Some of the time; Less than half of the time; More than half of the time; Most of the time; All of the time. |
| Subcomponent | Felt calm & relaxed last 2 weeks (WHO) | Over the last two weeks, I have felt calm and relaxed.  The answer choices are the following: At no time; Some of the time; Less than half of the time; More than half of the time; Most of the time; All of the time. |
| Subcomponent | Felt active & vigorous last 2 weeks (WHO) | Over the last two weeks, I have felt active and vigorous.  The answer choices are the following: At no time; Some of the time; Less than half of the time; More than half of the time; Most of the time; All of the time. |
| Subcomponent | Woke up fresh & rested last 2 weeks (WHO) | Over the last two weeks, I woke up feeling fresh and rested.  The answer choices are the following: At no time; Some of the time; Less than half of the time; More than half of the time; Most of the time; All of the time. |
| Subcomponent | Daily life filled with things that interest last 2 weeks (WHO) | Over the last two weeks, my daily life has been filled with things that interest me.  The answer choices are the following: At no time; Some of the time; Less than half of the time; More than half of the time; Most of the time; All of the time. |
| Subcomponent | Felt unable to control important things last month (Cohen) | In the last month, how often have you felt that you were unable to control the important things in your life?  The answer choices are the following: Never; Almost never; Sometimes; Fairly often; Very often |
| Subcomponent | Felt confident about ability to handle personal problems last month (Cohen) | In the last month, how often have you felt confident about your ability to handle your personal problems?  The answer choices are the following: Never; Almost never; Sometimes; Fairly often; Very often |
| Subcomponent | Felt confident things were going your way last month (Cohen) | In the last month, how often have you felt confident that things were going your way?  The answer choices are the following: Never; Almost never; Sometimes; Fairly often; Very often |
| Subcomponent | Felt difficulties were piling up could not overcome them last month (Cohen) | In the last month, how often have you felt difficulties were piling up so high that you could not overcome them?  The answer choices are the following: Never; Almost never; Sometimes; Fairly often; Very often |
|  |  |  |
| **Secondary Outcome** | **Handwashing action** | Share of adult household members who were observed washing their hands at any juncture, measured via structured observations.  The considered junctures are the following:  Before obtaining water from a wide-mouthed storage container;  Before cutting or preparing food;  Before serving food;  Before eating;  Before feeding child under 5;  Before breastfeeding child;  After defecation;  After toileting;  After cleaning child post- toileting |
|  |  |  |
| **Secondary Outcome** | **Handwashing with soap/ash** | Share of adult household members who were observed washing their hands with soap/ash at any juncture, measured via structured observations.  The considered junctures are the following:  Before obtaining water from a wide-mouthed storage container;  Before cutting or preparing food;  Before serving food;  Before eating;  Before feeding child under 5;  Before breastfeeding child;  After defecation;  After toileting;  After cleaning child post- toileting |
|  |  |  |
| **Secondary Outcome** | **School attendance** | Number of days child aged 6 to 18 years old attended school in past week, based on responses to “How many days has this child attended school in the past week?”  Children who were not enrolled in school were coded as zero. |
| **Secondary Outcome** | **Water point has water** | 1 if water point provides water  0 Otherwise  (Locked water points during survey are not considered) |
|  |  |  |
| **Secondary Outcome** | **WP Coliforms MPN/100mL** | Water quality at point of collection (village water source) is Most Probable Number (MPN) in 100 mL as defined for [Aquagenx](https://www.aquagenx.com/cbt-ectc/) CBT EC+TC MPN water quality testing kits and follow WHO standards. For nondetects, we substitute half the lower detection limit. |
|  |  |  |
| **Secondary Outcome** | **HH Coliforms MPN/100mL** | Water quality at point of use (Drinking water stored in the household) is Most Probable Number (MPN) in 100 mL as defined for [Aquagenx](https://www.aquagenx.com/cbt-ectc/) CBT EC+TC MPN water quality testing kits and follows WHO standards. For nondetects, we substitute half the lower detection limit. |
|  |  |  |
| **Secondary Outcome** | **Weight for age** | Weight for age z score for children under 5.  The sample does not include observations with implausible z-scores. |
|  |  |  |
| **Secondary Outcome** | **Weight for length** | Weight for length z score for children under 5.  The sample does not include observations with implausible z-scores. |
|  |  |  |
