# Supplementary material for: Effects of a community-driven water, sanitation, and hygiene intervention on diarrhea, child growth, and local institutions: A cluster-randomized controlled trial in rural Democratic Republic of Congo
Source: PLoS Med. 2025 Mar 6;22(3):e1004524. doi: 10.1371/journal.pmed.1004524 (PMC11884671; doi:10.1371/journal.pmed.1004524)
Supplement: S2 Text — (DOCX) [file pmed.1004524.s010.docx]

**PREANALYSIS PLAN**

**for**

**The People’s Water:
A Randomized Control Trial of a Community-Driven Water, Sanitation, and Hygiene Program in the Democratic Republic of Congo**

March 2020

| Aidan Coville  Research Department  The World Bank | Kevin Croke  School of Public Health  Harvard University | Eric Mvukiyehe  Research Department  The World Bank | John Quattrochi  Dept. of Public Health  Simmons University |
| --- | --- | --- | --- |

**Abstract**

*Since 2008, the Government of the DRC launched a national program known as National Healthy Villages and Schools (in French Villages et Ecoles Assainis, VEA), a water and sanitation program financed by DFID and implemented by UNICEF and the Government of DRC’s Ministry of Public Health and Ministry of Primary, Secondary, and Professional Education. The VEA’s main objectives are to support communities’ improved access to water, hygiene, and sanitation services and practices through the construction of water and sanitation infrastructure in villages and schools, local community involvement, and a village certification mechanism. This study uses a cluster-level randomized controlled trial to evaluate the causal impact of the VEA program across 332 rural villages implemented in the later phase of the program (throughout 2019). It involves experimentally varying the provision of the VEA program to a set of villages to examine the causal impacts on water access, infrastructure development, availability of sanitation services, knowledge of hygiene practices, and child health. Program impacts will be ascertained on a wide-range of key outcomes of interest, including water quantity and quality; health behavior/knowledge, time use, child health, and school attendance.*

**Keywords:** Community-Driven Water, Sanitation and Hygiene**; rural communities; conflict settings; cluster-randomized control trial; Democratic Republic of Congo.**

# **Introduction**

Rural WASH is inadequate in many developing countries, especially those affected by fragility, conflict and violence (FCV), with important implications for human welfare. This is the case in the Democratic Republic of Congo (DRC)— home to approximately 80 million people, including 7% of the world’s extreme poor. Seventy percent of the population is rural, and many residents have experienced protracted instability and violent conflict. The DRC ranked 176^th^ in the Human Development Index in 2015, with a life expectancy of 59.1 years and an adult literacy rate of 77%. The under-5 mortality rate is 104 per 1,000 live births (118 in rural areas). Only 31% of rural Congolese use an improved water source and only 29% use improved sanitation facilities (UNICEF/WHO JMP 2015). 59% of rural households spend more than 30 minutes (on each round trip) to collect drinking water.

Against this backdrop, in 2008, the Government of the DRC launched a national program known as the “National Healthy Village & Schools Programme” (in French, *Villages et Ecoles Assainis-VEA*), a Water and Sanitation (WASH) program financed by DFID and implemented by UNICEF and the Government of DRC’s Ministries of Public Health, and Primary, Secondary, and Professional Education. The VEA’s main objectives are to support improved access to water, hygiene and sanitation (WASH), especially in rural areas. The VEA supports the construction of water and sanitation infrastructure in villages and schools, facilitates local community involvement in management of this infrastructure, conducts behavior change activities, and then formally certifies communities (as “Healthy Villages” or “Healthy Schools”) when they achieve a set of water and sanitation norms. Since 2008, approximately 6,500,000 people in several thousand villages have been reached with WASH services through the program (UNICEF 2017). The program is currently in Phase 2 (2013-2018) which, to support this impact evaluation, will offer an extended VEA programme to an additional 240 villages and 60 schools in 5 provinces (Kongo Central, Kasai, Kasai Central, South Kivu, North Kivu).

Given the strategic importance of the VEA as one of DFID’s biggest investments in the WASH sector, accounting for over 50 percent of its DRC budget in this sector, and the largest WASH program implemented by UNICEF globally, the Development Impact Evaluation Unit (DIME) of the World Bank’s Research Group is partnering with DFID and UNICEF to conduct a rigorous evaluation study, which will provide scientific evidence of the effects of the VEA program. Specifically, this evaluation study entails both an impact evaluation that seeks to ascertain the program’s impacts on key outcomes of interest, and a process evaluation that focuses on specific program elements (e.g., coordination among different stakeholders, sustainability, and the extent of community involvement) that are crucial to the implementation strategy. The study will also comprise in-depth qualitative research to unpack processes and mechanisms that underlie the functioning of the VEA program. This study will present robust evidence to improve policy decisions pertaining to WASH policies and programs in the DRC and beyond.

This pre-analysis plan focuses on the impact evaluation component of the study, with a specific focus on the outcomes that will be measured in a midline survey. Given the short time between program completion and fielding of the midline survey, we primarily measure program implementation in this survey. The endline data collection will likely include additional outcomes and measurement strategies, including an expanded sample to increase power on key development outcomes which will be specified at a later stage and documented in an addendum to this pre-analysis plan. This pre-analysis plan focuses on the midline survey, which means that we focus on a more limited set of outcomes, primary relating to water access and program implementation, than in the eventual endline survey, which will be a large survey with more detailed measurement of health outcomes.

# **Hypotheses/Evaluation Questions**

The primary hypotheses this evaluation seeks to investigate pertain to the impact of the VEA program on key outcomes of interest, including: (i) access to higher quality water and improved sanitation; and (ii) reduced burden of collecting and carrying water (measured by time used for collection) especially for women and girls.

More formally, we propose to test the following primary hypotheses using the midline data:

*Hypothesis 1: VEA will improve households’ water quality*

*Hypothesis 2: VEA will improve sanitation facilities that households use*

*Hypothesis 3: VEA will increase the quantity of water that households use*

*Hypothesis 4: VEA will reduce the time spent collecting water*

In addition, there are several secondary hypotheses of interest implied by the theory of change. They address potential health and education outcomes and the mechanisms by which the inputs from VEA could produce the desired outcomes. Improvements in the water sources and in the point-of-use are related to the reduction of diarrhea, but effects can be larger if purification treatments are sustained over time (Shimamura et al. 2017). As for the participation of community members in leading the WASH projects, a study of the Community-Led Total Sanitation (CLTS) in Ethiopia and Ghana found that community engagement-led training of local leaders reduced open-defecation (Crocker et al. 2011). Another evaluation of community involvement in WASH projects in Bangladesh found that handwashing after defecation significantly improved among men, but hygiene practices before preparing, serving or eating food remained extremely low (Johnston et al. 2009). Taken together, these studies call for finding and testing the best ways to assure that the program can lead to more effective WASH governance, more sustained functionality of WASH infrastructure, reduced cost of water, and more effective community involvement including via sustained behavior change, such that the program can translate into water and sanitation gains for individuals and households. Thus, this study will also investigate the following hypotheses^[[1]](#footnote-1)^:

*Hypothesis 5*: *VEA will increase the number of functional water points in a village*

*Hypothesis 6*: *VEA will reduce the cost of water*

*Hypothesis 7*: *VEA will increase satisfaction with access to water*

*Hypothesis 8*: *VEA will increase the probability that a village has a committee that is responsible for water*

*Hypothesis 9*: *VEA will increase the frequency with which health zone representatives visit a village*

*Hypothesis 10*: *VEA (via the behavioral change encouragement activities) will increase the frequency of improved health behaviors*

Program impacts of water and sanitation interventions have a mixed track record. Particularly for sanitation, the current experimental evidence has a wide variety of results, including many null results for diarrhea, which has often been identified as a primary outcome for these studies (Whittington et al, 2020). Reflecting on this, the research takes a cautious approach to measuring impacts. In the midline we will run exploratory analysis on health and education outcomes driven by the following hypotheses:

*Hypothesis 10: VEA will increase children’s school attendance, conditional on their enrollment*

*Hypothesis 11: VEA will reduce the incidence of diarrhea for U5 children*

The longer-term follow up survey will expand the set of outcome measures, which will be described in detail in the updated PAP.

# **Study Design**

## Treatment and Control Groups

Our goal is to estimate the causal effects of the core VEA program with a randomized control trial. The unit of treatment is the village; thus, a straightforward randomization would be at the village level. However, VEA villages can be quite close to one another, raising the possibility of spillover effects from treatment villages to control villages (e.g. people in control villages accessing new water points in treatment villages). Therefore, we grouped villages into clusters. We considered any villages within 2.5 km of each other (with distance determined “as-the-crow-flies”) to be part of the same cluster. Therefore, all clusters have at least 2.5 km between them. We relax this rule in South Kivu, where density is greater and we seek to include naturally occurring villages, rather than the smaller unit of VEA sub-villages, in the sample. In cases where naturally occurring/administrative villages are less than 1 km from each other, we aggregate these into a single village cluster.

UNICEF generated and shared with the research team a list of 403 candidate villages based on the established criteria for the program: the village is located in a secure and accessible Health Area that is not already served by the WASH Consortium, the Health Area staff are dynamic and interested in participating, and there is a problem of diarrhea, cholera, and/or malnutrition.^[[2]](#footnote-2)^ Thirty-four of these villages were already in process (in Kasai Central), leaving 369 eligible villages in five provinces: Kongo Central, Kasai, Kasai Central, South Kivu, and North Kivu. Based on the rules above, we grouped those villages into 124 clusters. North Kivu had only three clusters (covering 30 villages), so we decided that it was not logistically efficient to work there. That left us with 121 clusters in four provinces and 339 villages.

We randomized these into 50 treatment clusters (containing 146 treatment villages) and 71 control clusters (containing 187 control villages). Six villages were randomly dropped to ensure UNICEF target numbers were met, while maintaining treatment and control balance.^[[3]](#footnote-3)^ We stratified randomization to ensure that the treatment and control groups are balanced with respect to their (i) province and (ii) number of villages that make up a cluster. Stratified randomization ensures that the treatment and control groups will be statistically indistinguishable from each other with respect to these characteristics, thereby avoiding bias in treatment effect.

Treatment clusters of villages will receive the status quo VEA intervention, as described above. Control clusters of villages will not receive any intervention from VEA. Data collection procedures will be identical in the two groups, as described below.

Additional discussions were held regarding the possibility of overlaying the school intervention as part of the impact evaluation. Due to the fact that the schools had been pre-selected in each province, this was not possible. There is, however, some possibility that the school intervention is differentially implemented in proximity to treatment and control villages which could threaten the validity of the results, particularly on schooling-related outcomes.

The IE team will work closely with UNICEF and local implementing partners to ensure accurate GPS and implementation tracking information is included in the planned data collection exercises to ensure that, if imbalance in proximity to treatment schools does occur, we will be able to adequately control for this in the analysis.

## Survey rounds and timeline

We plan to conduct two major rounds of household surveys: (i) a midline survey at least 12 months after the start of implementation: surveys of village leadership, WASH infrastructure, and selected households in treatment and control clusters to capture medium-term effects on main implementation outcomes such as access to improved water sources, quality of water, access to and use of improved latrines and diarrhea; and (ii) an endline survey at least 24 months after the start of implementation: surveys of village leadership, WASH infrastructure, and selected households in treatment and control clusters to capture longer-term effects.

We did not carry out a baseline prior to the rollout of project activities due to operational and timeline constraints.

## Sample size and power calculations

In the midline we conduct a village-level survey with the village chief and/or water committee chair in each of the 332 study villages, as well as a household survey from a random sample of 4 households in each village. The objective is to have enough power to estimate initial impact of program implementation, while also helping to finetune the sample size requirements to measure development impacts in the longer-term follow up survey (including generating estimates on the standard deviation and intra-cluster correlation of some of selected health and education measures).

For the midline power calculations, we focus on a primary outcome – whether the household uses an improved water source as their primary source of water. Since the program focuses on villages that have poor water access, we anticipate the level in the control group to be quite low. We also expect a high intra cluster correlation since households in a given village will likely be using the same available water sources as their main water sources.

For these calculations, we limit the risk of Type I errors (false positives) to 5% (alpha = 0.05) and Type II errors (false negatives) to 20% (beta = 0.8). We use the Joint Monitoring Program (JMP)/UNICEF estimate for DRC nationally as an upper bound for improved water access. This is 42%. Since this is a cluster-RCT, we also need to estimate the intra-cluster correlation which is not possible with the available data. Instead we use a conservatively high level of 0.7 given the common infrastructure used in many villages. With 4 households sampled from each village, a 22 percent (or 8-percentage point) increase in access to improved water could be detected using the 121 clusters. Given the large changes anticipated in these operational outcomes, this is expected to be within expectations for the program

# **Key Outcomes of Interest**

This study will measure VEA program impact on a set of primary outcomes outlined in the stated goals of the VEA program based on the midline data, including: (i) access to higher quality water; (ii) use of improved sanitation; and (iii) reduced burden of carrying water (in time spent carrying water). In addition, it will investigate a set of secondary outcomes including willingness to pay for water, improved health behaviors, child illness, hygiene practices, and school attendance, and local water and sanitation governance, among others.

In the midline survey, several outcomes will be measured indirectly. For example, one primary outcome is water quality, which will be measured indirectly, by capturing whether the water source is considered “improved” or not (i.e. boreholes are considered improved while unprotected springs or surface water sources are not). However, in the endline round of data collection we plan to measure water quality directly with microbiological testing. These direct measures are still being developed and field-tested and will be submitted through an addendum to the pre-analysis plan before the endline data collection.

Similarly, capturing biomarkers (e.g. anthropometrics, stool samples, anemia blood tests) is the most objective and accurate way to measure health impacts, particularly in settings like WASH interventions where the treatment group cannot be blinded to the intervention and often suffers from social desirability bias where respondents receiving the intervention over-report positive health impacts. However, biomarkers are costly and logistically challenging to implement. Since a number of recent studies have found limited impacts in biomarker measures because of small changes in intermediate outcomes, we propose a phased approach.

Some intermediate, self-reported outcomes will be measured in the first (midline) follow-up and, reflecting on these intermediate measures and funding availability, a decision will be made on whether the second follow-up will include biomarkers. The latter could include hemoglobin and stool sample helminth tests to check for gut infections and anemia, anthropometrics to test for stunting and wasting due to poor nutrient absorption, and cortisol tests to measure overall well-being and stress associated with improvements in living conditions. We will measure the key survey-based outcomes of interest through surveys of the children’s main caregiver, using DHS, MICS, and LSMS questionnaires as main references.

**Table 1. Key outcomes of interest**

| **Outcome Type*** | **Outcome Name** | **Definition** | **Survey** | **Analysis level** |
| --- | --- | --- | --- | --- |
| Primary | Water access | Total time spent collecting water yesterday | Household | Household |
| Primary | Water quality | Primary source of drinking water is improved source (JMP definition) | Household | Household |
| Primary | Water quantity | Total amount of water collected yesterday | Household | Household |
| **Primary** | Sanitation access | Household uses an improved latrine (JMP definition) | Household | Household |
| **Secondary** | Health behavior / Knowledge | Handwashing practices (primarily of caregiver) based on handwashing with soap + water or ash at critical junctures  Knowledge: Caregiver knows how and when to wash hands; what causes diarrhea  Sanitation practices: Cleanliness of household area and latrine (presence of flies and fecal matter); open defecation; observed indicators of toilet use –worn pathway, presence of water; improvements to latrine; disposes of child feces safely (JMP definition)  Water storage practices: has a clean pot for water that is covered | Household | Household |
| **Secondary** | Child health | Percentage of children under 5 years old with diarrhea/fever/coughing in the past two weeks; water-related mortality of children | Household | Child |
| **Secondary** | School attendance | Number of days present per child in past week, conditional on child being enrolled in school | Household | Child |
| **Secondary** | Functional water points | Number of functional improved water points in cluster; number and length of breakdowns; time to fill 5-liter jerry can; year-round availability | Village | Community |
| **Secondary** | Financial cost of water | Household water use expenses in last week | Household | Household |
| **Secondary** | Satisfaction | Satisfaction with access to water | Household | Household |
| **Secondary** | Water governance | Is there a committee that is responsible for water; size of committee and inclusion of minorities; has a water maintenance system; time since visited by health zone; satisfaction with health zone, health area and central govt. | Village | Village |

*Other outcomes not in this list may be analyzed on an exploratory basis.

# **Estimation**

We will separately estimate the main impacts of VEA on the outcomes listed above, using the following basic specification:

$y_{ihvc}= +\beta_{1}T_{c}+ \boldsymbol{\gamma X}_{ihvc}+\boldsymbol{\delta Z}_{ihvc}\boldsymbol{+}\varepsilon_{i}$ (1)

where $y_{ihvc}$ is the outcome of interest for respondent *i* in household *h* in village *v* in cluster *c* at the follow-up survey, defined above. $T_{i}$ is the treatment indicator that takes value 1 for clusters that were randomly assigned to participate in VEA (“treatment clusters”) and 0 for otherwise (“control clusters”). $\boldsymbol{X}_{ihvc}$ represents a set of strata-specific dummies where strata are based on province and number of villages in the cluster, which will equal 1 if the household falls in that stratum, and 0 otherwise. $\boldsymbol{Z}_{ihvc}$ is a vector of baseline covariates included in the analysis. Specifically we include gender and age (month) dummies for all <5 child health outcomes, but do not include covariates for other outcomes. $\boldsymbol{\gamma}$ and $\boldsymbol{\delta}$ are vectors of associated strata and covariate coefficients respectively. $\varepsilon_{i}$ is an idiosyncratic error term. Our main parameter of interest is $\beta_{1}$, the intention-to-treat effect (ITT). Standard errors will be clustered at the randomization (village cluster) level.

Our primary outcomes comprise four different measures. Our secondary outcomes comprise a broader range of variables, particularly the health behavior outcome set. To reduce the number of statistical tests and reduce the probability of false positives (Type I errors), when an outcome space is comprised of multiple variables (such as the health behavior outcomes) we combine measures into an index following (Kling, Liebman, and Katz 2007). For our four primary outcomes we use the (Benjamini, Krieger, and Yekutieli 2006) method to control the false discovery rate (FDR) and report adjusted p-values for these primary outcome measures. We do not apply this to secondary (exploratory) variables.

# For the balance test we include:

- VEA school in village cluster
- Type of roof (improved roof= 1 if roof is finished roofing (i.e. *Metal, Wood, Calamine/Cement fiber Ceramic tiles, Cement, or Roofing shingles*)
- Wall (improved walls = 1 if walls are “finished walls”)
- Floor (improved floor= 1 if floor is “finished floor”)
- ~~Sex of respondent~~
- Household size
- Religion (Catholic = 1)
- Mother tongue
- Marital status 🡺 binary variable for married

# **Subgroup And Spillover analysis**

1. As with many interventions of this kind, it is likely that the VEA program may work differently for different socioeconomic groups. For instance, the program may have relatively larger effects on poorer/vulnerable communities, as compared to communities that are relatively affluent, and it may have larger effects on women than on men. Thus, in addition to estimating the main treatment effects, we will investigate potential heterogeneity of the VEA program based on several pre-treatment (or time-invariant) characteristics related to the VEA program or the local context. Our primary interest for sub-group analysis is to explore gender differences in time collecting water. We have additional exploratory interest in:
2. Province (each of the four provinces).
3. Distance to territory capital (a binary indicator for above or below the median distance for the province).
4. Education (a binary indicator for above or below median formal education of the household head).
5. Conflict history (a binary indicator for above or below the median number of conflict events experienced in a village prior to program onset).

Each subgroup analysis will cover the set of primary outcomes described in Section 4.

We model heterogeneous treatment effects by the following equation:

$y_{ihvc}= \alpha_{s}+\beta_{1}T_{c}+\beta_{2}T_{c}* Z_{c} + {\beta_{3}Z}_{c}+ \varepsilon_{i}$ (2)

where $y_{ihvc}$ is the outcome of interest for respondent *i* in household *h* in village *v* in cluster *c.* at the follow-up survey, defined above. $T_{i}$ is the treatment indicator that takes value 1 for clusters that were randomly assigned to participate in VEA (“treatment clusters”) and 0 for otherwise (“control clusters”). $Z_{c}$ is a vector of baseline characteristics (e.g. gender) by which we are interested in heterogeneous treatment effects. $\beta_{2}T_{c}* Z_{c}$ represents an interaction between the treatment and those characteristics. $\alpha_{s}$ captures stratum-specific fixed effects, where strata are based on province and number of villages in the cluster. $\varepsilon_{i}$ is an idiosyncratic error term. Our main parameter of interest is $\beta_{2}$.

In South Kivu, some treated clusters may be just over 1 km from control clusters. To probe for potential spillovers, we follow Null *et al*. (2018) and test whether primary outcomes were the same in control households with more versus fewer households receiving interventions within a 2 km radius.^[[4]](#footnote-4)^ This will allow us to assess whether spillovers may be a concern for the primary analysis where we focus on direct effects.

# **Operationalization of key outcome variables**

This section lists the main outcome variables that will be used in the data analysis to measure the short-term impact of the VEA program, grouped in main outcome categories. We fully pre-specify our primary outcome measures and provide details on question inputs that will be used to construct secondary measures.

**Primary outcomes**

- *Water access:* Amount of time spent collecting water in minutes by the household in the previous day, calculated by summing up all trips x trip times in roster (Q. HH–A.4). Question asks: “How long did it take to fetch the water and come back on this trip, after leaving the house (including traveling there, waiting, and returning)?” for each trip.
- *Water quality*: What is the main source of drinking water for members of your household? (Q. HH–A.1). Binary outcome = 1 if response = 1 (piped into dwelling); 2 (piped into plot); 3 = piped/public tap); 5 (tube well or borehole); 7 (protected spring). Outcome is assigned 0 for all other response. (basic improved water source definition as per JMP)
- *Water quantity:* Total amount of water collected the day before interview in liters (Q. HH–A.4). Number of jerry cans multiplied by the liters in each jerry can, summed over all trips taken in previous day
- *Sanitation access:* Where do members of your household usually go to defecate (Q. HH-F.1). Binary outcome = 1 if response = 1 (flush/pour to piped sewer); 2 (flush/pour to septic tank; 3 (flush/pour to pit latrine), 6 (ventilated improved pit latrine); 7 (pit latrine with slab); 8 (composting latrine). Outcome is assigned 0 for all other responses. This follows the basic improved sanitation definition as per JMP.

**Secondary and exploratory outcomes**

**Water quality and access**

- Total number of water points in the villages (VL–B.1)
- Total number of functional water points in the villages (VL–B.4)
- Total number of functional improved water points in the villages (VL–B.4.2)
- Total number of breakdowns in functional water points in past 6 months (VL–B.4.11)
- Total length of breakdowns in functional water points in past 6 months (VL–B.4.12)
- Total length of last breakdown in functional water points before reparation (VL–B.13)
- Number of trips to collect water conducted the day before interview by women and children (Q. HH–A.4)
- Distance of primary water source from center of the village (Q. VL–B.4.7)
- Time to fill a 5-liter jerry can at primary water source, in minutes (Q. HH–A.7)
- Average water availability year-round (Q. VL–B.4.22)
- Community perception of water quality (Q. VL–4.26)

**Child health**

- Under 5 child suffered from diarrhea in the past 2 weeks – self-reported by mother (Q. HH–0.21)
- Under 5 child suffered from fever in the past 2 weeks – self-reported by mother (Q. HH–0.22)
- Under 5 child suffered from coughing in the past 2 weeks – self-reported by mother (Q. HH–0.23)
- Cumulative index of overall under 5 child health (Q. HH-0.21–0.23)
- Mortality in past 18 months? 🡪 Child deceased due to water-related health reasons in past 18 months (HH–0.14–0.17)

**School attendance**

- School-aged child usually attends school (Q. HH-0.19)
- Number of days school-aged child was present at school in past week, conditional on child being enrolled in school (Q. HH-0.20)

**Health behavior and knowledge**

*Handwashing practices*

- Proportion of caregivers using soap/ashes for handwashing (Q. HH-E.1)
- Proportion of caregivers washing hand appropriately (Q. HH–E.2)
- Cumulative index of handwashing practices (Q. HH–E.2)
- Proportion of households who own a handwashing device (Q.HH-F.13)

*Handwashing and hygiene knowledge*

- Proportion of caregivers who knows the causes of diarrhea (Q. HH–E.3)

*Sanitation practices*

- Improved defecation practices (Q. HH­–F.1–F.3)
- Frequency of latrine cleaning/maintenance (Q. HH–F.4–F.9)
- Improvements to latrines implemented in past 18 months (Q. HH–F.12)
- Safe disposal of youngest child feces, conditional on having a child (Q. HH–F.14)
- Cleanliness of household area, e.g. observed presence of flies, mosquitos (Q. HH-F.15, F.16)
- Frequency of household area cleaning/maintenance (Q. HH–E.5)
- Garbage disposal (Q. HH–E.4)

*Water storage practices*

- Proportion of households that own a pot for water storage (Q. HH-E.6)
- Water from the pot is clean (Q. HH-E.7)
- Water from the pot is covered (Q. HH-E.8)
- Pot has clean cup (Q. HH-E.9)

**Financial cost of water**

- Household pays to collect water (Q. HH–A.8)
- Household expenses for water use in last week (Q. HH–A.16)
- Household usually pays village leaders to collect/use water (Q. HH-A.17)

**Community satisfaction with water**

- Household satisfied with access to water (Q. HH-D.1)
- Household satisfied with water management (Q. HH-B.5)

**Water Governance**

- Presence of a committee/association/group or single individuals that report to the community about the management of water services (Q. VL–B4.27)
- Presence of a WASH committee responsible for water (Q. VL–ID.15)
- Size of WASH/water committee (Q. VL–I.11.1, VL–H.31)
- Inclusion of minorities in WASH/water committee (Q. VL–I.11.3, VL–H.32.3)
- Presence of a water maintenance system (Q. VL–B.6, HH–B.6, HH–B.7)
- Time since most recent visit by health zone representative (Q. VL–Min of F.10-F.14)
- Community satisfied with health zone administration (Q. VL–F.1)
- Community satisfied with health area administration (Q. VL–F.1)
- Community satisfied with national central government administration (Q. VL–F.1)

# **Appendix**: Randomization Annex

UNICEF provided DIME with a set of 403 georeferenced villages across the 5 districts of Kongo Central, Kasai, Kasai Central, South Kivu and North Kivu, where villages were defined based on the EVA program definition. From this dataset, 34 villages from Kasai Central were already indicated to have been assigned to receive the program, leaving a universe of 369 program villages to conduct the randomization. The main requirements that were imposed on the randomization were:

1. The number of treatment villages needed to match exactly with the number of UNICEF target villages for the program in each province.
2. Due to concerns of possible spillovers, the randomization needed to cluster villages deemed to be too close to each other.

*What were the clustering rules used?* In rural provinces (Kasai, Kasai Central and Kongo Central) any village within 2.5km (as the crow flies) of another village would be considered as part of the same cluster. This was discussed as an appropriate distance to avoid meaningful spillovers since it would imply an approximately 45-minutes one-way journey to the program water source on average which far exceeds the SDG definition of improved water requiring that a round trip take 30 minutes or less. The more densely populated urban areas (South and North Kivu) require a modified clustering rule to avoid all villages joining a single cluster. Here the procedure is to cluster all program villages that belong to the same administrative villages into single clusters (i.e. consider the administrative village rather than the program village as the unit of randomization). However, some administrative villages lie very close to one another. As an additional level of clustering, we then combine all administrative villages that lie within 1km of each other^[[5]](#footnote-5)^.

Once clusters are identified, we randomize clusters into treatment and control groups, stratifying by province and cluster size to ensure balance. In all provinces except Kasai Central, each cluster is given equal probability of being selected for treatment or control. In Kasai Central, we increase the probability of being selected into the control group proportionally to reflect the fact that only 16 out of 81 villages from the sampling frame were required to be assigned to receive treatment.

Since randomization is based on clusters but UNICEF targets are based on villages, it is not possible to force the randomization to select the exact number of UNICEF villages targeted without introducing potential bias. Instead we compare the number of UNICEF target villages per province to the number of treatment villages selected after randomization. In cases where the number of treatment villages is larger than the target, we randomly drop an equal number of program villages from the largest control and treatment clusters until targets are met. For Kongo Central this meant dropping 2 villages and for Kasai this meant dropping 4 villages split equally from the largest treatment and control clusters.

The result of the randomization selection is summarized in the below table:

| **Province** | **Total sampling frame of villages received** | **Treatment villages (clusters)** | **Control villages (clusters)** | **Villages dropped** |
| --- | --- | --- | --- | --- |
| **Kongo Central** | 40 | 20 (9) | 18 (9) | 2 |
| **Kasai** | 98 | 50 (20) | 44 (19) | 4 |
| **Kasai Central** | 81 | 16 (8) | 65 (29) | 0 |
| **South Kivu** | 120 | 60 (13) | 60 (14) | 0 |
| **Total** | **339** | **146 (50)** | **187 (71)** | **6** |
| ***North Kivu*** | *Excluded due to lack of clusters (3) from the province* | | | |

1. Arguably, the proposed mechanisms or channels cannot be identified causally within the current study design. Rather, these will be investigated through qualitative research. [↑](#footnote-ref-1)
2. Unicef. Logigramme Villages et Ecoles Assainis. Guide de Sélection des Villages et des Ecoles. Version février 2014. Fichier .ppt. [↑](#footnote-ref-2)
3. A more detailed description of the sampling and randomization can be found in the Randomization Annex. [↑](#footnote-ref-3)
4. We note that this is a robustness check rather than a formal test to try find spillovers. [↑](#footnote-ref-4)
5. In practice, we find the centroid of all GPS points of program villages that are clustered into an administrative unit and compare this centroid between clusters to assess how close each administrative village is. In some cases, GPS points are clearly inaccurate (e.g. indicating that a program village lies in the water). In these cases, we remove that village GPS from the centroid calculations and rely on the other program villages that are part of the same cluster. [↑](#footnote-ref-5)
