# Supplementary material for: Effects of a community-driven water, sanitation, and hygiene intervention on diarrhea, child growth, and local institutions: A cluster-randomized controlled trial in rural Democratic Republic of Congo
Source: PLoS Med. 2025 Mar 6;22(3):e1004524. doi: 10.1371/journal.pmed.1004524 (PMC11884671; doi:10.1371/journal.pmed.1004524)
Supplement: S3 Text — (DOCX) [file pmed.1004524.s011.docx]

**PRE-ANALYSIS PLAN**

**for**

**Health and governance effects of a Randomized Control Trial of a Community-Driven Water, Sanitation, and Hygiene Program in the Democratic Republic of Congo**

March 2023

| Aidan Coville  Development Impact Evaluation Department  The World Bank | Kevin Croke  School of Public Health  Harvard University | Eric Mvukiyehe  Department of Political Science  Duke University | John Quattrochi  Global Infectious Diseases Program  Georgetown University |
| --- | --- | --- | --- |

**Abstract**

**Background:** In 2008, the Government of the DRC launched a national program known as National Healthy Villages and Schools (in French, “Villages et Ecoles Assainis”, VEA), a water and sanitation program financed by FCDO and implemented by UNICEF and the Government of DRC’s Ministry of Public Health and Ministry of Primary, Secondary, and Professional Education. The VEA’s main objectives are to support communities’ improved access to water, hygiene, and sanitation services through the construction of water and sanitation infrastructure in villages and schools, and to promote community mobilization around hygienic water and sanitation practices.

**Methods**: This study uses a cluster-level randomized controlled trial to evaluate the causal impact of the VEA program across 332 rural villages implemented throughout 2019. Communities were experimentally allocated to the VEA program in order to examine the causal impact of the program on water and sanitation access, water supply governance, management, and maintenance, knowledge attitudes and practices related to hygiene, and child health and nutrition outcomes.

**Discussio**n: A midline survey demonstrated that the VEA program positively affected water and sanitation access, and led to self-reported behavior change. This study will use data from an endline survey to examine program effects on primary outcomes of child health and nutrition, and water and sanitation governance, as well as secondary outcomes such as water access and quality, and childrens’ school attendance.

**Keywords:** Community-Driven Water, Sanitation and Hygiene**; rural communities; conflict settings; cluster-randomized control trial; Democratic Republic of Congo.**

# **Introduction**

Rural WASH is inadequate in many developing countries, especially those affected by fragility, conflict and violence (FCV), with important implications for human welfare. This is the case in the Democratic Republic of Congo (DRC)— home to approximately 80 million people, including 7% of the world’s extreme poor. Seventy percent of the population is rural, and many residents have experienced protracted instability and violent conflict. The DRC was ranked 176^th^ in the Human Development Index in 2015, with a life expectancy of 59.1 years and an adult literacy rate of 77%. The under-5 mortality rate is 104 per 1,000 live births (118 in rural areas). Just 31% of rural Congolese use an improved water source and only 29% use improved sanitation facilities (UNICEF/WHO JMP 2015). 59% of rural households spend more than 30 minutes on each round trip to collect drinking water.

Against this backdrop, in 2008, the Government of the DRC launched a national program known as the “National Healthy Village & Schools Programme” (in French, *Villages et Ecoles Assainis-VEA*), a Water and Sanitation (WASH) program financed by the UK Foreign and Commonwealth Development Office^[[1]](#footnote-1)^ (FCDO) and implemented by UNICEF and the Government of DRC’s Ministries of Public Health, and Primary, Secondary, and Professional Education. The VEA’s main objectives are to support improved access to water, hygiene and sanitation (WASH), especially in rural areas. The VEA supports the construction of water and sanitation infrastructure in villages and schools, facilitates local community involvement in management of this infrastructure, conducts behavior change activities, and then formally certifies communities (as “Healthy Villages” or “Healthy Schools”) when they achieve a set of water and sanitation norms. Since 2008, approximately 6,500,000 people in several thousand villages have been reached with WASH services through the program (UNICEF 2017). The program evaluated by this study was VEA’s Phase 2 (2013-2018) which offered an extended VEA programme to 240 villages and 60 schools in 5 provinces (Kongo Central, Kasai, Kasai Central, South Kivu, North Kivu). Funding for the VEA program was discontinued by FCDO in 2022.

Before its discontinuation, VEA was one of FCDO’s biggest investments in the WASH sector, accounting for over 50 percent of its DRC budget in this sector, and the largest WASH program implemented by UNICEF globally. Given the importance of the program, the Development Impact Evaluation Department (DIME) of the World Bank is partnering with FCDO and UNICEF to conduct a rigorous evaluation study, which will provide evidence of the effects of the VEA program. Specifically, this evaluation study entails both an impact evaluation that seeks to ascertain the program’s impacts on key outcomes of interest, and a process evaluation that focuses on specific program elements (e.g., coordination among different stakeholders, sustainability, and the extent of community involvement) that are crucial to the implementation strategy. The study will also comprise in-depth qualitative research to unpack processes and mechanisms that underlie the functioning of the VEA program. This study will present robust evidence to improve policy decisions pertaining to WASH policies and programs in the DRC and beyond.

A first study, using a midline survey fielded a median of 5 months after program implementation, measured implementation fidelity and examined the effect of the program on water and sanitation access, water governance, self-reported hygiene practices, school attendance, water collection burden, and satisfaction with WASH services. This analysis showed that VEA resulted in large increases in access to improved water sources and improved sanitation sources, but did not have significant effect on time to collect water or quantity of water collected (primary outcomes). VEA also resulted in improvements in satisfaction with water, with water governance, and on self-reported handwashing and sanitation indices. There was no effect on the financial cost of water, water storage, school attendance, or a water quality and access index (secondary outcomes).

This pre-analysis plan is for research to be conducted using the evaluation endline survey, which will leverage a larger sample to measure VEA’s effect on key development outcomes such as child health and nutrition, and community governance of WASH resources. It will also provide a longer term assessment of the VEA program’s impacts on intermediate indicators such as access to improved water and sanitation.

# **Hypotheses/Evaluation Questions**

The primary hypotheses relate to the impact of the VEA program on child health, and on community water supply management. The primary outcomes are (i) reduced diarrheal disease for children under 5; (ii) improved nutrition for children under 5; and (iii) improved governance of WASH resources.

The rationale for these outcomes is as follows. The VEA’s main objective is to support improved access to WASH in rural communities via a four- part intervention: provision of new or improved water infrastructure; provision of new or improved sanitation infrastructure; strengthening of village-level WASH institutions; and a behavior change campaign facilitated by external actors. The campaign comprises multiple mobilization and education events, and culminates when communities that meet a set of water and sanitation norms through VEA are formally certified as ‘Healthy Villages’.

The goal of VEA was therefore not just to finance the construction or improvement of new water and sanitation infrastructure, but also to change knowledge about hygiene and norms for healthy behavior, and to support the creation of more functional village institutions for management of WASH resources. Therefore, in addition to focusing on health and nutrition outcomes, we also study the functioning of village WASH governance.

More formally, we propose to test the following primary hypotheses:

*Hypothesis 1: VEA will reduce the frequency of diarrheal disease over preceding 7 days for children under 5*

*Hypothesis 2: VEA will improve nutrition of children under 5 (length-for-age Z scores)*

*Hypothesis 3: VEA will improve the functioning of village-level water governance institutions*

In addition, there are several secondary hypotheses of interest implied by the theory of change (see secondary outcomes in Table 1 and discussion below). They address children’s education outcomes which could be affected by improved water and sanitation (e.g. reduced absenteeism in school), as well as *mechanisms* by which the inputs from VEA could produce the desired health outcomes (such as improved water and sanitation access, improved water quality, and sanitation behavior change).

# **Study Design**

## Treatment and Control Groups

The study’s goal is to estimate the causal effects of the core VEA program, using a randomized control trial research design. The program’s unit of treatment is the village, therefore we begin with randomization at the village level. However, some modest refinements must be made to a basic cluster randomized design to account for features of this setting. The first modification is applied because VEA villages can be quite close to one another. This raises the possibility of spillover effects from treatment villages to control villages (e.g. people in control villages accessing new water points in treatment villages). Therefore, we grouped villages into clusters. We considered any villages within 2.5 km of each other (with distance determined “as-the-crow-flies”) to be part of the same cluster. Therefore, all clusters have at least 2.5 km between them. We relax this rule in South Kivu, where density is greater and we seek to include naturally occurring villages, rather than the smaller unit of VEA sub-villages, in the sample. In cases where naturally occurring/administrative villages are less than 1 km from each other, we aggregate these into a single village cluster.

UNICEF generated and shared with the research team a list of 403 candidate villages for this phase of the VEA program, based on the established criteria for the program: the village is located in a secure and accessible Health Area that is not already served by the WASH Consortium, the Health Area staff are dynamic and interested in participating, and there is a problem of diarrhea, cholera, and/or malnutrition.^[[2]](#footnote-2)^ Thirty-four of these villages already had program activities in process (in Kasai Central) before research activities began, leaving 369 eligible villages in five provinces: Kongo Central, Kasai, Kasai Central, South Kivu, and North Kivu. Based on the rules above, we grouped those 369 villages into 124 clusters. North Kivu had only three clusters (covering 30 villages), so we decided that it was not logistically efficient to work there. That left 121 clusters (339 villages) in four provinces.

The study team randomized these into 50 treatment clusters (containing 146 treatment villages) and 71 control clusters (containing 187 control villages). Six villages were randomly dropped to ensure UNICEF target numbers were met, while maintaining treatment and control balance.^[[3]](#footnote-3)^ We stratified randomization to ensure that the treatment and control groups are balanced with respect to (i) province and (ii) number of villages that make up a cluster. Stratified randomization ensures that the treatment and control groups will be statistically indistinguishable from each other with respect to these characteristics, likely increasing the precision of final estimates.

Treatment clusters of villages received the VEA intervention, as described above. Control clusters of villages did not receive any intervention from VEA. Data collection procedures will be identical in the two groups, as described below.

## Survey rounds and timeline

The full VEA evaluation includes two major rounds of household surveys: (i) a midline survey which was conducted in November/December 2019; and (ii) an endline survey planned in late 2022 and early 2023.^[[4]](#footnote-4)^ This PAP discusses analysis of data collected in the endline survey.

## Sample size and power calculations

The midline survey was comprised of a village-level survey with the village chief and/or water committee chair in each of the 332 study villages, as well as a household survey from a random sample of 4 households in each village. From 11 November 2019 to 23 December 2019, the survey team interviewed 1,312 households in 328 villages in 121 clusters in four provinces. This survey, which sampled 4 households from each village, was well powered to detect changes the most relevant intermediate outcome i.e. whether the household uses an improved water source as their primary source of water.

For the endline, we seek to power the trial to detect changes in diarrhea in children under 5, which had an intra-cluster correlation (ICC) of 0.09 in the midline survey. Therefore, we estimate that we would have 80% power to detect a decline in diarrhea prevalence of 8 percentage points^[[5]](#footnote-5)^, based on a sample of 10 households per village, who have on average 1.3 children under age 5 (approximately 36 observations per cluster, 4,317 child-level observations).

# **Key Outcomes of Interest**

This study will measure VEA program impact on a set of primary outcomes, which were defined based on the stated goals of the VEA program. These outcomes cover the domains of child health, child nutrition, and local water governance. In addition, in this study we will investigate a set of secondary outcomes including use of improved water sources and sanitation facilities, water quality at village and household sources, household-level hygiene practices, and school attendance.

*Primary outcomes: health and nutrition*

Health and nutrition outcomes for children under 5 will be captured directly in two ways.

First, the household survey will include a detailed battery of questions for the child’s caretaker which will ask about number of symptoms/illness episodes across symptoms related to diarrhea over the past 7 days (question include whether or not the child has experienced diarrhea, or has had 3 or more bowel movements within 24 hours, soft stools, or blood in stool at any point in the last 7 days).^[[6]](#footnote-6)^

In addition, we will collect anthropometric data, notably children’s height and weight. Using the height and weight measurements together with the child’s age and gender and comparing them against WHO Child Growth Standards, we will calculate z-scores for each child’s height for age (HAZ), weight for age (WAZ), and weight for height (WHZ), to measure nutrition-related conditions including stunting and underweight (Cashin and Oot, 2018)^[[7]](#footnote-7)^. We will report the height-for-age (HAZ) z- score as a primary outcome. Dichotomous measures of stunting (HAZ <-2 SD) and wasting (WHZ <-2 SD) prevalence will be secondary outcomes.

*Primary outcomes: WASH governance*

WASH governance outcomes will be measured via a series of questions about the existence and activities of village-level water and sanitation governing institutions such as WASH committees. We will create an index to capture summary measures of governance activities at village level. We also measure other aspects of WASH governance and sustainability as secondary outcomes, described further below.

*Secondary outcomes*

We have a large number of secondary outcomes. We may decide to describe effects on some secondary outcomes in separate manuscripts from the manuscript that presents effects on primary outcomes. We present them all here to avoid the ‘file drawer problem’ that will occur if researchers register multiple pre-analysis plans for a single project.

Key secondary outcomes include access to improved water and sanitation facilities, water quality at water points and in homes, improved hygiene knowledge and behaviors, measure of WASH governance, and children’s school absenteeism.

Healthy hygiene knowledge and behaviors will be measured through structured observation activities which will allow direct observation of handwashing and sanitation practices in respondents’ homes.

Access to improved water and sanitation will be captured based on self-report: whether respondents reported that their main water source is considered improved or not following the Joint Monitoring Program standard definitions (i.e. boreholes are considered improved while unprotected springs or surface water sources are not); cost of water, and number of liters collected by each household daily.

We will measure water quality directly with microbiological testing. We will test quality for water samples collected (i) at each of the water points used by members of each village, and (ii) at the containers where water is stored at the household level for a subset of 6 households per village on average. Based on midline data, we estimate about 697 to 1,000 samples coming from village-level water points and about 2,000 samples coming from household level water-storage containers. Testing will be done with Aquagenx CBT EC+TC Most Probable Number (MPN) Kit.

We will use survey-based measures focused on subjective performance of local WASH institutions: respondent views about fairness of selection of water governance entity, perception of fair treatment, confidence in entity’s management of money, confidence in response to breakdowns, confidence in management, overall satisfaction. We will also use direct observation of water point functionality and reported length of breakdowns via a water point observation and water committee and village leader survey.

**Table 1. Key outcomes of interest**

| **Outcome Type*** | **Outcome Name** | **Definition** | **Survey** | **Analysis level** |
| --- | --- | --- | --- | --- |
| Primary | Diarrhea | Prevalence of diarrhea in last 7 days, children under 5 (provider reported) | Household | Individual |
| Primary | Length-for-age | Length for age z score for children under 5 | Household | Individual |
| Primary | Institutions | Index comprised of:  1. Presence of water committee 2. Frequency of meeting 3. Time spent on non-maintenance activities 4. Has a maintenance plan 5. Tracks health conditions 6. Tracks hygiene and sanitation | Village survey | Village |
| Secondary | Committee performance | Water committee performance (length of water point breakdowns) | Village leader; water point | Village |
| Secondary | Governance perceptions | Water governance perception index (fairness of selection of water governance entity, perception of fair treatment, confidence in managing money, confidence in response to breakdowns, confidence in management, overall satisfaction) | Household | Individual |
| Secondary | Water quality | Thermotolerant coliforms per 100 mL water | Water point | Water point |
| Secondary | Water quality | Thermotolerant coliforms per 100 mL water | Household | Household |
| Secondary | Water access | Primary source of drinking water is improved source (JMP definition) | Household | Household |
| Secondary | Water access | Household water use expenses in last week | Household | Household |
| Secondary | Water access | Time spent collecting water | Household | Household |
| Secondary | Sanitation access | Household uses an improved latrine (JMP definition) | Household | Household |
| Secondary | Observed handwashing | Handwashing practices of caregiver based on handwashing with soap and water or ash at critical junctures, measured via structured observations | Structured observations | Household |
| Secondary | Health behavior and knowledge | Self-reports of:  Knowledge: Caregiver knows how and when to wash hands; what causes diarrhea  Sanitation practices: Cleanliness of household area and latrine (presence of flies and fecal matter); open defecation; observed indicators of toilet use –worn pathway, presence of water; improvements to latrine; disposes of child feces safely (JMP definition)  Water storage practices: has a clean pot for water that is covered | Household | Household |
| Secondary | School attendance | Number of days present per child in past week, conditional on child being enrolled in school | Household | Child |
| Secondary | Life satisfaction and self-esteem | Summary index of 11 questions | Household | Individual |
| Secondary | Psychological well-being | Summary index of 9 questions on well-being and stress in last 2-4 weeks | Household | Individual |
| Secondary | Wasting | Prevalence of wasting, children under 5 (weight for height z score) | Household | Child |
| Secondary | Underweight | Prevalence of underweight, children under 5 (weight for age z score) | Household | Child |

*Other outcomes not in this list may be analyzed on an exploratory basis.

# **Estimation**

We will separately estimate the main impacts of VEA on the outcomes listed above, using the following basic specification:

$y_{ihvc}= +\beta_{1}T_{c}+ \boldsymbol{\gamma X}_{ihvc}+\boldsymbol{\delta Z}_{ihvc}\boldsymbol{+}\varepsilon_{i}$ (1)

where $y_{ihvc}$ is the outcome of interest for respondent *i* in household *h* in village *v* in cluster *c* at the follow-up survey, defined above. $T_{i}$ is the treatment indicator that takes value 1 for clusters that were randomly assigned to participate in VEA (“treatment clusters”) and 0 for otherwise (“control clusters”). $\boldsymbol{X}_{ihvc}$ represents a set of strata-specific dummies where strata are based on province and number of villages in the cluster, which will equal 1 if the household falls in that stratum, and 0 otherwise. $\boldsymbol{Z}_{ihvc}$ is a vector of baseline covariates included in the analysis. Specifically we include gender and age (year) dummies for all <5 child health and nutrition outcomes, but do not include covariates for other outcomes. $\boldsymbol{\gamma}$ and $\boldsymbol{\delta}$ are vectors of associated strata and covariate coefficients respectively. $\varepsilon_{i}$ is an idiosyncratic error term. Our main parameter of interest is $\beta_{1}$, the intention-to-treat effect (ITT). Standard errors will be clustered at the randomization (village cluster) level.

Our primary outcomes comprise three different measures. Our secondary outcomes comprise a broader range of variables, particularly the health behavior outcome set. To reduce the number of statistical tests and reduce the probability of false positives (Type I errors), when an outcome space is comprised of multiple variables (such as the health and hygiene behavior) we combine measures into an index following (Kling, Liebman, and Katz 2007).

# Although we do not have survey data collected prior to program implementation, we will also examine whether there are systematic differences between the treatment and control on traits which are determined prior to program start (i.e. markers of identity such as language or religion, as well as relatively fixed assets such as housing quality), namely:

- Type of roof (improved roof= 1 if roof is finished roofing (i.e. *Metal, Wood, Calamine/Cement fiber Ceramic tiles, Cement, or Roofing shingles*)
- Wall (improved walls = 1 if walls are “finished walls”)
- Floor (improved floor= 1 if floor is “finished floor”)
- Religion (Catholic = 1)
- Mother tongue
- Marital status (married=1)

# **Subgroup analysis**

As with many interventions of this kind, it is likely that the VEA program may work differently for different populations or in different settings. Thus, in addition to estimating the main treatment effects, we will investigate potential heterogeneity of the VEA program based on several pre-treatment (or time-invariant) characteristics related to the VEA program or the local context, namely:

1. Province (each of the four provinces, for all primary outcomes).
2. Gender (for health and nutrition outcomes only)

We model heterogeneous treatment effects by the following equation:

$y_{ihvc}= \alpha_{s}+\beta_{1}T_{c}+\beta_{2}T_{c}* Z_{c} + {\beta_{3}Z}_{c}+ \varepsilon_{i}$ (2)

where $y_{ihvc}$ is the outcome of interest for respondent *i* in household *h* in village *v* in cluster *c.* at the follow-up survey, defined above. $T_{i}$ is the treatment indicator that takes value 1 for clusters that were randomly assigned to participate in VEA (“treatment clusters”) and 0 for otherwise (“control clusters”). $Z_{c}$ is a vector of baseline characteristics (e.g. gender) by which we are interested in heterogeneous treatment effects. $\beta_{2}T_{c}* Z_{c}$ represents an interaction between the treatment and those characteristics. $\alpha_{s}$ captures stratum-specific fixed effects, where strata are based on province and number of villages in the cluster. $\varepsilon_{i}$ is an idiosyncratic error term. Our main parameter of interest is $\beta_{2}$.

# **Operationalization of key outcome variables**

This section lists the main outcome variables that will be used in the data analysis to measure the short-term impact of the VEA program, grouped in main outcome categories. We fully pre-specify our primary outcome measures and provide details on question inputs that will be used to construct secondary measures.

***Primary outcomes***

1. **Diarrhea**

We will code a child as positive for diarrhea if, in the 7-day recall section, the respondent answers

1. Yes to ‘diarrhea’ , or

2. Yes to ‘three or more…” AND ‘watery or soft stool’, or

3. Yes to blood in the stool

We will use two-week recall as a sensitivity analysis. In this approach, we will code a child as positive for diarrhea if the respondent answers

1. Yes to ‘diarrhea’, or
2. Yes to ‘blood in the stool’

We did not ask about other symptoms for two week recall.

1. **Child length-for-age**

Child's recumbent length, standardized to Z-scores using the WHO 2006 growth standards

1. **WASH institutions index**

Using the ‘greedy’ version of the Kling, Liebman, and Katz (2004) mean effects index, we will combing the following measures from the village leaders survey:

- 1. Yes to either of the following:
     1. G.1. Is there a <b>Water, Sanitation, and Hygiene (WASH) committe</b> active in your community?
     2. G.2. Is there <b>any committee, association, group or individual that is active on water issues</b> (e.g. controls access to water points, collects fees for water use, organises repairs, etc) in your community?
  2. G.18. How often does the committee meet? [coded as ordinal variable]
  3. J.14. In this village, is there a community maintenance action plan?
  4. G.35. What is the average amount the committee spends in total <b>each month</b> for <i>water-related activities</i> (excluding maintenance)? If this variable has >10% missing, we will instead use the sum of the following for all committee members:
     1. G.15.6. How many hours per week does ${comm_member_name} work on the committee?
  5. J.1. In your village, do you usually keep track of the community’s health conditions (for example, the number of people who are ill due to drinking non-potable water or their knowledge of hygiene practices)?
  6. J.2. In your village, do you usually keep track of hygiene and sanitation practices in this community (such as households’ defecation practices and hand-washing behavior)?

***Secondary outcomes***

*Committee performance*

- Mean length of water point breakdowns

*Governance perceptions*

- overall satisfaction with WASH governance: (HHB4)
- fair process for selection (HH.B7); does it treat you fairly (B8)

How well does the water committee respond to breakdowns (HH.B9)

How well does the water committee manage money? (HHB10)

Are you confident they would try to solve the problem? (HHB11)

*Water quality, water point*

Thermotolerant coliforms per 100 mL water

*Water quality, household*

Thermotolerant coliforms per 100 mL water

*Water source*

Primary source of drinking water is improved source (JMP definition)

*Water expenses*

Household water use expenses in last week

*Water collection time*

Total time spent collecting water by all household members

*Sanitation quality*

Household uses an improved latrine (JMP definition)

*Observed handwashing*

Percentage of observed events where adult hands were washed adequately (both hands washed, with soap or ash) prior to event. [Events are defined as using toilet, preparing food, eating food, or changing infant diaper/cleaning child post-toilet]

*Health behavior and knowledge*

Self report: Handwashing and hygiene knowledge

- Proportion of caregivers who knows the causes of diarrhea (Q. HH–E.3)

Sanitation practices

- Improved defecation practices (Q. HH­–F.1–F.3)
- Frequency of latrine cleaning/maintenance (Q. HH–F.4–F.9)
- Improvements to latrines implemented in past 18 months (Q. HH–F.12)
- Safe disposal of youngest child feces, conditional on having a child (Q. HH–F.14)
- Cleanliness of household area, e.g. observed presence of flies, mosquitos (Q. HH-F.15, F.16)
- Frequency of household area cleaning/maintenance (Q. HH–E.5)
- Garbage disposal (Q. HH–E.4)

Water storage practices

- Proportion of households that own a pot for water storage (Q. HH-E.6)
- Water from the pot is clean (Q. HH-E.7)
- Water from the pot is covered (Q. HH-E.8)
- Pot has clean cup (Q. HH-E.9)

*School attendance*

Number of days present per child in past week, conditional on child being enrolled in school

*Weight-for-height*

Weight-for-height z score, children under 5

*Weight-for-age*

Weight-for-age z score, children under 5

*Life satisfaction and self-esteem*

MH1. All things considered, how satisfied are you with your life as a whole?

"MH4. Rosenberg's Self-Esteem Scale: Below is a list of statements dealing with your general feelings about yourself. Please indicate how strongly you agree or disagree with each statement.

I will make certain statements for you. That means, you are going to act as if it is you who are talking to me. You will listen to me and tell me to what extent you: Strongly agree, Agree, Disagree, or Strongly disagree with these statements about you.

It's about what you feel and think, there really is no ""right"" or ""wrong"" answer."

MH4.1. I feel that I am a person of worth, at least on an equal plane with others.

MH4.2. I feel that I have a number of good qualities.

MH4.3. All in all, I am inclined to feel that I am a failure.

MH4.4. I am able to do things as well as most other people.

MH4.5. I feel I do not have much to be proud of.

MH4.6. I take a positive attitude toward myself.

MH4.7. On the whole, I am satisfied with myself.

MH4.8. I wish I could have more respect for myself.

MH4.9. I certainly feel useless at times.

MH4.10. At times I think I am no good at all.

*Psychological well-being*

"MH2. WHO questions on well-being: Please, indicate for each of the five statements which is closest to how you have been feeling over the last two weeks. Notice that higher numbers mean better well-being.

I will make certain statements for you. That means, you are going to act as if it is you who are talking to me. You will listen to me and tell me how this statement fits with how you are feeling or thinking by responding with: All the time, Most of the time, More than half the time, Less than half the time, Some of the time, or At no time.

It's about what you feel and think, there really is no ""right"" or ""wrong"" answer."

MH2.1. Over the last two weeks, I have felt cheerful and in good spirits.

MH2.2. Over the last two weeks, I have felt calm and relaxed.

MH2.3. Over the last two weeks, I have felt active and vigorous.

MH2.4. Over the last two weeks, I woke up feeling fresh and rested.

MH2.5. Over the last two weeks, my daily life has been filled with things that interest me.

Enumerator say: The next questions will ask about how you've felt over the last month.

"MH3. Cohen stress scale: The questions in this scale ask you about your feelings and thoughts during THE LAST MONTH.

It's about what you feel and think, there really is no ""right"" or ""wrong"" answer."

MH3.1. In the last month, how often have you felt that you were unable to control the important things in your life?

MH3.2. In the last month, how often have you felt confident about your ability to handle your personal problems?

MH3.3. In the last month, how often have you felt confident that things were going your way?

MH3.4. In the last month, how often have you felt difficulties were piling up so high that you could not overcome them?

# Attrition

We will apply attrition corrections if the treatment indicator (with attrition as dependent variable) is significant at p<0.05. We will use the Kling-Liebman sensitivity bounds approach (Kling and Liebman 2004). This approach presents treatment effect estimates for a wide range of possible values of missing data. This approach demonstrates the extent to which selective attrition changes treatment effect estimates. For example, if the program is shown to reduce diarrheal incidence by 10%, this approach would show how this parameter would change under a range of assumptions about diarrheal incidence in the attrited group.

For the subset of observations for which we have previous survey information from the midline, we can also use inverse probability weighting, in which we model non-response as a function of midline household characteristics (e.g. age, education household size, wall, floor, and roof material, province-cluster stratum) and re-weight non-attrited respondents accordingly.

# **Appendix**: Randomization Annex

UNICEF provided DIME with a set of 403 georeferenced villages across the 5 districts of Kongo Central, Kasai, Kasai Central, South Kivu and North Kivu, where villages were defined based on the EVA program definition. From this dataset, 34 villages from Kasai Central were already indicated to have been assigned to receive the program, leaving a universe of 369 program villages to conduct the randomization. The main requirements that were imposed on the randomization were:

1. The number of treatment villages needed to match exactly with the number of UNICEF target villages for the program in each province.
2. Due to concerns of possible spillovers, the randomization needed to cluster villages deemed to be too close to each other.

*What were the clustering rules used?* In rural provinces (Kasai, Kasai Central and Kongo Central) any village within 2.5km (as the crow flies) of another village would be considered as part of the same cluster. This was discussed as an appropriate distance to avoid meaningful spillovers since it would imply an approximately 45-minutes one-way journey to the program water source on average which far exceeds the SDG definition of improved water requiring that a round trip take 30 minutes or less. The more densely populated urban areas (South and North Kivu) require a modified clustering rule to avoid all villages joining a single cluster. Here the procedure is to cluster all program villages that belong to the same administrative villages into single clusters (i.e. consider the administrative village rather than the program village as the unit of randomization). However, some administrative villages lie very close to one another. As an additional level of clustering, we then combine all administrative villages that lie within 1km of each other^[[8]](#footnote-8)^.

Once clusters are identified, we randomize clusters into treatment and control groups, stratifying by province and cluster size to ensure balance. In all provinces except Kasai Central, each cluster is given equal probability of being selected for treatment or control. In Kasai Central, we increase the probability of being selected into the control group proportionally to reflect the fact that only 16 out of 81 villages from the sampling frame were required to be assigned to receive treatment.

Since randomization is based on clusters but UNICEF targets are based on villages, it is not possible to force the randomization to select the exact number of UNICEF villages targeted without introducing potential bias. Instead we compare the number of UNICEF target villages per province to the number of treatment villages selected after randomization. In cases where the number of treatment villages is larger than the target, we randomly drop an equal number of program villages from the largest control and treatment clusters until targets are met. For Kongo Central this meant dropping 2 villages and for Kasai this meant dropping 4 villages split equally from the largest treatment and control clusters.

The result of the randomization selection is summarized in the below table:

| **Province** | **Total sampling frame of villages received** | **Treatment villages (clusters)** | **Control villages (clusters)** | **Villages dropped** |
| --- | --- | --- | --- | --- |
| **Kongo Central** | 40 | 20 (9) | 18 (9) | 2 |
| **Kasai** | 98 | 50 (20) | 44 (19) | 4 |
| **Kasai Central** | 81 | 16 (8) | 65 (29) | 0 |
| **South Kivu** | 120 | 60 (13) | 60 (14) | 0 |
| **Total** | **339** | **146 (50)** | **187 (71)** | **6** |
| ***North Kivu*** | *Excluded due to lack of clusters (3) from the province* | | | |

1. At the time of the evaluation, the program was funded by DFID (the UK’s Department for International Development) which was later merged with the Foreign and Commonwealth Office to form what is now referred to as FCDO. We use FCDO throughout this note to refer to DFID and FCDO. [↑](#footnote-ref-1)
2. Unicef. Logigramme Villages et Ecoles Assainis. Guide de Sélection des Villages et des Ecoles. Version février 2014. Fichier .ppt. [↑](#footnote-ref-2)
3. A more detailed description of the sampling and randomization can be found in the Randomization Annex. [↑](#footnote-ref-3)
4. Three rounds of mobile phone surveys were conducted in 2020-2021 focused on COVID-19 knowledge and behavior. These surveys are not discussed here. [↑](#footnote-ref-4)
5. This holds if the prevalence of diarrhea in children under 5 in the treatment group is at most 21%, given that the midline prevalence of diarrhea in children under 5 in the control group was 29%. [↑](#footnote-ref-5)
6. Later in the survey, we also ask about diarrhea with a 14 day recall period, in order to benchmark our estimates to the DHS survey in DR Congo, which uses this recall period. [↑](#footnote-ref-6)
7. Kristen Cashin and Lesley Oot. 2018. Guide to Anthropometry: A Practical Tool for Program Planners, Managers, and Implementers. Washington, DC: Food and Nutrition Technical Assistance III Project (FANTA)/ FHI 360. [↑](#footnote-ref-7)
8. In practice, we find the centroid of all GPS points of program villages that are clustered into an administrative unit and compare this centroid between clusters to assess how close each administrative village is. In some cases, GPS points are clearly inaccurate (e.g. indicating that a program village lies in the water). In these cases, we remove that village GPS from the centroid calculations and rely on the other program villages that are part of the same cluster. [↑](#footnote-ref-8)
