# Supplementary material for: Effects of a community-driven water, sanitation, and hygiene intervention on diarrhea, child growth, and local institutions: A cluster-randomized controlled trial in rural Democratic Republic of Congo
Source: PLoS Med. 2025 Mar 6;22(3):e1004524. doi: 10.1371/journal.pmed.1004524 (PMC11884671; doi:10.1371/journal.pmed.1004524)
Supplement: Attachment — Submitted filename: Response to reviewers.docx [file pmed.1004524.s012.docx]

**We thank the editors and reviewers for their thoughtful and constructive comments. We addressed them below, in bold.**

-----------------------------------------------------------

Comments from the academic editor:

The academic editor was supportive of your work. However, she commented that the reviews were robust and agreed that the concerns raised required addressing in full. She is looking forward to reviewing a revised manuscript.

-----------------------------------------------------------

Comments from the reviewers:

Reviewer #1: This manuscript reports on the results from a cluster-randomized trial of a national WASH program in the DRC. The results are consistent with other recent large studies of WASH interventions in terms of the limited impact on outcomes. A major strength of the study is the length of follow-up - 3.5 years. Some methodological details need clarification as described below. Overall, I don't feel the manuscript is sufficiently novel to warrant publication in PLOS Medicine.

Minor comments:

1. Introduction - what is VEA? The Healthy Villages and Schools program?

**Yes, VEA stands for “Village et Ecole Assainis” or “Healthy Villages and Schools” in English. We have replaced all instances of VEA with “Healthy Villages and Schools.”**

2. Methods - was distance between villages from the village center? Or village boundary?

**Distance was calculated from village centers (centroids). We clarified this on line 143: “We considered any villages within 2.5 km of each other (using Euclidean distance between village centroids) to be part of the same cluster.”**

3. Methods - the meaning of this is unclear "Six villages were randomly dropped to ensure UNICEF target numbers were met"

**We clarified this in the Randomization and Masking section. “We block randomized by province and number-of-villages-per-cluster (12 blocks total; see Table S1). Since randomization was based on clusters but the implementing organization’s operational targets were based on villages, it was not possible to force the randomization to select the exact number of villages targeted without introducing potential bias. Instead we compared the number of target villages per province to the number of treatment villages selected after randomization. In cases where the number of treatment villages was larger than the target, we randomly drop an equal number of program villages from the largest control and treatment clusters until operational targets were met. We dropped 2 villages in Kongo Central and 4 villages in Kasai. We also dropped one control village in Kasai due to a coding error. This left 146 villages in 50 clusters in the intervention group, and 186 villages in 71 clusters in the control group.”**

4. Methods - for stratified randomization, was number of villages in the cluster categorized in some way?

**Yes, we created three strata within each province, with strata based on the number of villages per cluster (see Table S1). In this way we maintained similar-sized clusters in both treatment and control groups.**

5. In general, abbreviations are confusing and need to be defined at first use

**We apologize for the lack of clarity. We have defined all abbreviations or spelled them out.**

6. Methods - its not clear what data collection was for the intervention program vs. for this research study and it would be helpful to clarify that. More clarity of the timing of measurement of each outcome would also be helpful

**All of the data collection was for the research study. The surveys were specific to the evaluation. In ‘Study Design’, we now state “With direction from the study investigators, Innovative Hub for Research in Africa (IHfRA) was responsible for data collection. No data used in this study was collected by intervention implementers.”**

**Data collection for the 3.5 year follow-up was conducted between November 24, 2022 and February 10, 2023. The first sentence of ‘Results’ now reads “Of the 1,312 respondents in 328 villages interviewed for the 5-month follow-up, 1,133 (86%) were re-interviewed at the 3.5 year follow-up, between November 24, 2022 and February 10,2023.”**

7. Methods - more details on the timing and samples collected for the water testing is needed

**We now provide more details in the ‘Outcomes’ section. As part of the water testing, there were two samples. The first sample consisted of all water sources commonly used in each study village. The second sample consisted of samples from water stored in households for drinking purposes.**

**The Aquagenx CBT EC+TC MPN Kit was used for the exercise. It simultaneously detects and quantifies E. coli (EC) and Total Coliform (TC) bacteria in a 100 mL sample.**

**More details of the testing kits and methods can be found** [**here**](https://www.aquagenx.com/wp-content/uploads/2023/11/MPN-CBT-ECTC-Instructions-DrinkingWater-20231114.pdf)**.**

8. Statistical analyses - how could the sample size be determined based on results after follow-up started?

**The reviewer is correct that the number of clusters (groups of villages) was determined at the time of randomization, in 2018. We calculated sample size for the 3.5 year follow-up in terms of the number of households per village that we would interview, taking the number of clusters as fixed based on the initial study design from 2018. The number of clusters was determined based on a) number of villages that the program implementer (UNICEF) was responsible for conducting the program in; and b) the number of geographic clusters which could be created from this number of villages, while following a clustering rule to avoid spillovers from treatment to control villages. The clustering rule was that all villages within 2.5 km Euclidean distance would be considered a single cluster (this was altered to a 1 km Euclidean distance in the more densely populated South Kivu province).**

**The number of households per cluster for the survey was then determined prior to survey implementation in 2023. We designed our household sampling to power the study to detect changes in diarrhea in children under 5, which had an intra-cluster correlation (ICC) of 0.09 in the midline survey. Therefore, we estimated that we would have 80% power to detect a decline in diarrhea prevalence of 8 percentage points, based on a sample of 10 households per village.**

9. What was the rationale behind interviewing some individuals that had been interviewed at 5 months and some that weren't? were these considered differently in the analysis?

**We wanted to increase our sample size from the 5-month follow-up to the 3.5 year follow-up, therefore we interviewed six more households in each village, in addition to the four households from the 5-month follow up. We did not consider these two groups differently in this analysis.**

10. Discussion - it would be better to focus on the explanation of the findings that is most likely rather than listing all the options. Here there was no reduction in pathogen exposure which may be the most likely explanation?

**Thank you for this comment, we agree that it’s important for the discussion to be focused on the most important findings of the study (effects on primary outcomes), and we agree that failure to reduce pathogen exposure is the most likely explanation. We deleted the third paragraph in the discussion which considered other explanations. The second paragraph discusses how the null effects on anthropometrics are in line with previous trials and how they are likely driven by persistent fecal contamination. The third paragraph focuses on how pathogen exposure remains high in the intervention group.**

11. Table 1 - the standard deviation for binary variables is not interpretable. Can you instead report the village prevalence median and interquartile range (to handle the geographic clustering)

**We appreciate the point about standard deviations for binary variables being uninterpretable, or at least not providing any new information once the mean is known, and we acknowledge the reviewer’s point that it is important to account for geographic clustering. To account for geographic clustering, we now regress each variable on intervention group, using the same functional form as for our estimates of the intervention effect, namely: fixed effects for randomization strata (province and cluster size) with standard errors clustered by cluster (group of villages). Table 2 contains the results.**

12. Table 2- for diarrhea the "mean" is a prevalence? It is unclear how to interpret the standard deviation for diarrhea prevalence. This also applies to binary outcomes in Table 3

**Yes, the mean is a prevalence. We have re-labed Tables 2 & 3 (now Tables 3 & 4) to make that clearer, and we no longer report the SD, since it does not provide any new information once the mean is known.**

Reviewer #2: General comments

In this article, Quattrochi and colleagues report results from a large, cluster randomized trial of a national-level Water, Sanitation, and Handwashing (WASH) program in rural DRC, where they measured effects on child diarrhea, linear growth, and community WASH indicators. I commend the authors on the completion of this important study, which provides important (if disappointing), null results regarding the effect of the program on child health. The trial was conducted in a study population with extremely high burden of diarrhea (42% of children <5 y had diarrhea in the control group in the previous 7d) and linear growth faltering (mean z = -2.2 in the control group). Although not measured, the study communities presumably experience very high levels of enteric pathogen infection and likely could benefit from improved WASH conditions.

I have some concerns about lack of detail in the outcome measurement methods, which I have listed below in major comments. Presuming those details can be clarified the trial did have good precision to rule out even small differences between groups that are clinically relevant (difference in diarrhea prevalence +/- 4%, difference in length-for-age z of +/- 0.14). Therefore, if measurement approaches were reasonable I would consider it an informative (null) trial. I hope my comments are constructive.

MAJOR comments

(1)

Page 14, Paragraph starting: "The study team randomized these into 50 intervention clusters (containing 146 intervention villages) and 71 control clusters (containing 187 control villages)."

These details about randomization seem out of place, as they should go in the Randomization and Masking section on the following page 15.

**We agree, and we have moved this information to the Randomization and Masking section.**

Regarding the randomization, it would be helpful if you could describe the randomization with a few more details. I can gather that you stratified the randomization by province and cluster size. However, it would be helpful for you to report the following.

* Number of strata in the randomization. Since cluster size (number of villages) is an integer count, were clusters matched exactly on size or within bands or nearest neighbor? If the latter, please specify the ranges used. Again, specific description of the number of provinces x cluster size strata would be helpful.

**We created three strata within each province, with strata based on the number of villages per cluster (see Table S1). In this way we maintained similar-sized clusters in both treatment and control groups. We clarified this in the Randomization and Masking section.**

* Then, please clarify if the randomization was simple randomization within strata or blocked. If blocked, were block sizes randomly permuted or fixed? Typically, blocking is recommended to preserve balance within strata so it would be helpful to understand these details (not the end of the world if it wasn't blocked).

**Within each stratum, we randomized 1:1 intervention:control in Kongo Central, Kasai, and South Kivu; in Kasai Central, we randomized 1:3 intervention:control due to budget constraints. We clarified this in the Randomization and Masking section.**

* for the 15 villages that were dropped due to "program constraints", was that done before the stratification and randomization, or after? If after, then it would undermine, slightly, the stratification by cluster size in those cases. Either way, it would be helpful to be clearer about these details.

**We misreported the number of villages dropped; apologies for this error. The correct number is 7. These were dropped after randomization: “In cases where the number of intervention villages was larger than the operational target, we randomly dropped an equal number of intervention villages from the largest control and intervention clusters until operational targets were met. We dropped 2 villages in Kongo Central and 4 villages in Kasai. We also dropped one control village in Kasai due to a coding error.”**

**The dropping of these villages also did not result in the clusters shifting across the threshold from “large” to “medium”, and so we would still have had the same distribution for cluster groups. As such, this should not have major effect on stratified randomization. We have updated the text to clarify this.**

(2)

Page 15, Procedures

It would be helpful if the authors could provide more detail about the dates of program implementation. The outcome measures are described as taking place 3.5 years after implementation, but it was unclear if this was from the start of activities or from the point when communities had progressed through all 8 steps described in the paper.

**In intervention villages, the median time between the completion of Healthy Villages Step 6 (construction of infrastructure) and data collection was 3.6 years (IQR = 3.4 to 3.7). Data collection was conducted between November 24, 2022 and February 10, 2023. The 5-month follow-up data collection was carried out between October 11 and December 23, 2019, which corresponded to a median of 5 months after program implementation in treatment villages.**

(3)

Page 17, Anthropometry outcomes

Please explain the rationale for a prioritizing anthropometric growth measurements among children ages 2-5 years old. Please provide detail about how the field team measured child anthropometry. Did the team follow a standard protocol? For example, did the team collect replicate measurements? Did they measure recumbent length amongst children <24 months? I noticed that the SDs of 1.6 for length-for-age (should it be called height-for-age since it includes children older than 24 months?) are quite high for a z-score. Typically, z-scores would have SD close to 1.0 in studies with very high quality anthropometric measurements.

**We prioritized the youngest child aged 2-5 years, followed by the eldest child aged 0-2 years, because we theorized that the effect of the intervention would be higher in children that were exposed for a greater length of time. However, children older than 3.5 years were born pre-intervention, and therefore the crucial early childhood period critical for long-run linear growth trajectories (i.e. stunting) would not be affected by the intervention. Our selection criteria reflect that tradeoff.**

**For the anthropometrics survey, we specifically followed protocols from the handbook titled “Recommendations for Data Collection, analysis and Reporting on Anthropometric Indicators in children under 5 Years Old” from the World Health Organization and the United Nations Children’s Fund (UNICEF), 2019. The book can be consulted here:** [**https://iris.who.int/bitstream/handle/10665/324791/9789241515559-eng.pdf?sequence=1&isAllowed=y.Licence:CCBY-NC-SA3.0IGO**](https://iris.who.int/bitstream/handle/10665/324791/9789241515559-eng.pdf?sequence=1&isAllowed=y.Licence:CCBY-NC-SA3.0IGO)**.**

**In addition, we have drawn from resources provided by the FANTA USAID program that can be consulted here:** [**https://www.fantaproject.org/tools/anthropometry-guide**](https://www.fantaproject.org/tools/anthropometry-guide)**.**

**As part of fieldwork, we conducted a blinded random re-measurement approach as a back-check, implemented by each team supervisor. Team supervisors were in charge of randomly selecting two households from all households where anthropometric measurements took place and re-measuring the child previously selected. In addition, among households where no child was measured, one household was randomly selected to confirm why no child was selected. This re-measurement exercise did not take place in Kongo Central (the province with the smallest sample in the study – villages represent about 11% of the study sample) due to timing constraints. However, rigorous high-frequency checks were conducted as the data was uploaded to ensure quality.**

**For children <24 months, recumbent length was measured.**

**Re: standard deviation of length-for-age measurements, Bekele et al (2020) report SDs from 16 studies that range from 0.9 to 2.19. (See Fig 2 from that paper below). This shows that, while measurement error can always be reduced, the SDs we report are in line with the published literature.**

(4)

Page 17, Diarrhea outcome

It would be helpful if you could clarify how and when were caregivers interviewed? In their home? A single interview, or in multiple, monthly visits? Was diarrhea defined by caregivers or by symptoms? If using a symptom-based definition, were caregivers asked about each symptom separately or all together?

**The female head of household was asked about all children living in the household. She was interviewed once, in her home. She was asked to respond yes or no to a list of symptoms in the last seven days.
In the SI, we note that we coded children as having had diarrhea if the respondent said:
1. Yes to ‘diarrhea’ , or
2. Yes to ‘three or more stools’ AND ‘watery or soft stool’, or**

**3. Yes to blood in the stool**

**The full list of symptoms (which includes symptoms of interest beyond diarrhea) is:
1. Fever**

**2. Diarrhea**

**3. Three (3) or more bowel movements within a 24-hour period**

**4. Water or soft stool (unformed)**

**5. Blood in the stool**

**6. Skin rash (anywhere on the body)**

**7. Constant cough**

**8. Congestion, runny nose**

**9. Panting, wheezing, difficulty breathing**

**10. Bruising, scrapes or cuts**

**11. Toothache, teething**

(5)

Page 17, Structured observation

Please explain the details of structured observation activities. E.g., Frequency? Duration? Key activities of focus? Coding? Standardization? This can be quite complex, and the description in the methods is scant.

**We revised the fourth paragraph in Outcomes to: “Structured observation of handwashing was done in four households per village, randomly selected from the ten selected for study participation. A research assistant spent two hours in each of these households, recording if handwashing occurred at critical junctures such as before preparing food (see SI for full list of junctures).”**

**In the SI, we state that the variable ‘handwashing action’ is defined as “Share of adult household members who were observed washing their hands at any juncture, measured via structured observations. The considered junctures are the following:
Before obtaining water from a wide-mouthed storage container;
Before cutting or preparing food;
Before serving food;
Before eating;
Before feeding child under 5;
Before breastfeeding child;
After defecation;
After toileting;
After cleaning child post- toileting**

**The structured observations involved a household observation period of approximately 2 hours in each of the 4 households in a village that were originally interviewed during midline survey. When these households were not found or not available, their replacement or a new endline household was considered. After obtaining consent from the respondent and confirming they would be present in the household for the next 2 hours, the enumerator would sit somewhere in the compound to observe household activities - typically scheduled for the morning. The activity took place before the caregiver interview to minimize the Hawthorne effect.**

(6)

Page 18. Water quality measurements.

The method used to measure water quality seems appropriate, but there are few details in the methods of what bacterial indicators were measured. In the results, I infer thermotolerant coliforms? These are very non-specific to human pathogens, but a reasonable, coarse indicator of fecal pollution (though from all mammals). Please provide additional details. Additionally, and this is relatively minor, but the results appear to be reported in terms of difference on the natural log scale, but for water quality indicators such as total coliforms it is more conventional to report them on the log base 10 scale. Civil engineers will immediately interpret a "half log reduction" on base 10, and improvements will not be as clear to most readers in the field if reported on the natural log scale.

**We confirm that results for contamination are reported on the log base 10 scale.**

MINOR comments

(7)

Does VEA represent an acronym? I could not find any explanation for what it means, except that this was an evaluation of the "VEA program".

**Yes, VEA stands for “Village et Ecole Assainis” or “Healthy Villages and Schools” in English. We have replaced all instances of VEA with “Healthy Villages and Schools.”**

(8)

Page 14

"…with distance determined 'as-the-crow-flies'"

I suggest avoiding colloquialisms. Do you mean Euclidean or great circle distance?

**We revised to “Euclidean distance”, which was the intended meaning.**

(9)

Page 14

"Stratified randomization ensures that the intervention and control groups will be in expectation statistically indistinguishable from each other with respect to these characteristics, likely increasing the precision of final estimates."

Gains in efficiency through stratification, though possible, are not due to improved balance within strata — gains depend on the correlation between outcomes in the two groups within strata. I suggest you simply omit this sentence, as you don't need to justify this element of the design, as there are many reasons to stratify beyond possible gains in precision.

**We accept the reviewer’s point and have accordingly deleted this sentence in the text.**

(10)

Page 15, Participants

It seems that children also participated in the study, not only women. Perhaps that should be explained here as well.

**We have updated the text to note that anthropometric data was collected on children when the caregiver provided consent for measurement.**

(11)

Page 18 Statistical Analysis

I was surprised to not see a sample size or detectable effect calculation for linear growth, a primary outcome for the trial. How could the trial have a primary outcome around which it was not designed? That seems a bit unconventional to me.

**In this case, we needed to pick a single primary outcome to determine sample size (since there is only a single sample size parameter that we could select) and, in consultation with our counterparts at the DRC Ministry of Health and UNICEF, we judged diarrhea prevalence to be the most relevant primary outcome given the nature of the intervention and the goals of the Ministry of Health for the program.**

(12)

Page 21

"Given the lack of an overall effect on diarrhea and length-for-age, we interpret the province-specific effects as statistical noise due to small sample sizes."

You could consider using less colloquial description than "statistical noise". It could be a chance finding or exploratory. This is a technical point, but the subgroup analyses were done by stratification and re-estimation of differences between groups. That is fine, but it fails to provide a formal test of effect modification on any scale (here, the additive scale is probably most appropriate). The authors could consider formal tests of interaction on the additive scale for each of their pre-specified subgroup analyses. Those are typically conducted using interaction terms in the linear regression models, and then a global test for model fit with and without the interaction term(s) (either Wald-type or likelihood-ratio-type tests).

**Thank you for this comment. We have now fitted models with interaction terms and conducted global tests for model fit, and now report those results in the last two paragraphs of the Results section, and in Tables S5 and S7.
“We also used interaction terms in linear models to test for effect modification on the additive scale (Table S5). Of the nine coefficients (three provinces, leaving out a reference, and three primary outcomes), two were statistically significant: in Kasai Central province, the results suggest that the intervention increased diarrhea prevalence relative to the treatment effect in the reference province (Kongo Central); in Kasai province, the results suggest that the intervention increased length-for-age. For both diarrhea and length-for-age, the Wald test rejects the null hypothesis that all province-by-intervention coefficients are zero at the 0.05 level. For the WASH institutions index, the Wald test fails to reject the null.**

**In the prespecified subgroup analysis of diarrhea and length-for-age by child sex, we find no difference in intervention effects by sex (see Supplementary Material Table S4). In linear models with interaction terms for intervention-by-sex, the coefficients are not statistically significant (Table S7).”**

(13)

Page 21

"We measured enteropathogens in water sources…"

Please re-word: you measured "fecal indicator bacteria" in water sources, which is quite different from enteric pathogens. Additionally, in this paragraph you suggest that the reason you may have not found differences in household water quality was due to the poor source water quality, but another reason could have been contamination between the source and stored water — widely documented in previous research. Finally, this paragraph ends with "More intensive WASH interventions may be necessary to prevent growth faltering.", which seems completely out of place in my opinion.

**We replaced “enteropathogens” with “fecal indicator bacteria.”
We added a sentence noting that in addition to poor source quality, fecal contamination could “also be linked to recontamination of water between collection and its ultimate use in the household.”**

**We deleted "More intensive WASH interventions may be necessary to prevent growth faltering."**

(14)

Table 1. It would be helpful to clarify in the caption or legend that these represent measures after 3.5 years of intervention, not at baseline

**The table is now captioned “Household and respondent characteristics by intervention group, at 3.5 year follow-up.”**

Reviewer #3: Introduction: I would recommend condensing and restructuring the introduction. You have a lot of good information; however, the global impact of community-based WASH interventions is is the majority of the intro and there is not any information to introduce the DRC context. It would be helpful to readers to link the global information to the country context in particular. For example, you mention "challenges of increasing access is particularly acute ... near armed conflict". This is an excellent segway into how this is pertinent to South Kivu, in particular.

**Thank you for this comment. We have added information to the Introduction section to link the global overview to the local context of the Democratic Republic of Congo.**

Please define VEA for readers

**VEA stands for “Village et Ecole Assainis” or “Healthy Villages and Schools” in English. We have replaced all instances of VEA with “Healthy Villages and Schools.”**

Typically at the end of the introduction the reader has a good grasp of what this article is aiming to describe and how this study will add to the current literature. However, I do not have have a clear grasp from the introduction what this manuscript is trying to accomplish. I would recommend more clearly stating your intent.

**We added this sentence to the end of the Introduction: “Our goal was to estimate the effect of Healthy Villages & Schools on diarrhea prevalence, child length-for-age, and WASH institutions.”**

Methods: I would recommend restructuring the study design section. I believe you can condense it into 2 paragraphs and be more concise with the wording. For example, it was not clear until the end of the second paragraph whether North Kivu was included in the analysis or not.

**Thank you for this comment; we have edited and condensed this section for greater clarity. We have also edited the trial flow diagram (Figure 1), including with regard to the point about North Kivu’s exclusion (pre-randomization) from the trial.**

I would recommend putting the 9 steps of the VEA in a table or flow chart

**Table 1 now contains the 9 steps of VEA (i.e. Healthy Villages and Schools).**

Did you collect information on access to food, antibiotics taken, any lab component or any other factors that could influence Z scores?

**Unfortunately, we were not able to collect this information.**

Results: I advise to be careful stating that the intervention had zero effect on XYZ, it has been 3.5 years since the intervention. What were the results from the 5-mo post intervention and how do they compare to the current analysis?

Thank you; we have edited the text to be much clearer that we do not find an effect at 3.5 years post-intervention. At 5 months post-intervention, we did not measure diarrhea nor height-for-age. We did, however, measure access to improved water and sanitation. We report those positive impacts in our previous publication. In the manuscript, we also compare these 5-month results to the 3.5 year results: “At five months post-intervention, the study team published results showing that access to improved water sources and improved sanitation facilities were higher in the intervention group. These improvements largely persisted to 3.5 years post-intervention, yet they did not have any measurable effect on diarrhea or growth faltering.”

i would recommend removing: The intervention had no effect on the psychological well-being index, the life satisfaction & selfesteem

index, or school attendance. completely. as this is not pertinent to the specific aims/objectives of this particular manuscript.

**While we agree with the reviewer that these outcomes may seem less directly related to the primary outcomes, an emerging literature in WASH has shown links between WASH interventions and socio-emotional outcomes. Motivated by this, we pre-registered these outcomes. Therefore we believe they should remain in the manuscript to reduce “file drawer bias” in literature when non-significant findings are not included in manuscripts.**

Discussion: I would recommend restructuring and focus on the lack of sustainability this intervention may have had. You also mention that the intervention water points were not significantly better than the control groups. This could be a huge transmission route for why the intervention appears to not have an effect. While the findings are very interesting and I believe could be a useful addition to the literature, I would be wary about claiming that the intervention had no effect on certain aspects. I recommend structuring the discussion on things the intervention succeeded may have succeeded in (i.e., more people seeking out a clean water source when available, or higher rates of hygiene) and then talk about where there are still gaps and how you would recommend future community-led interventions should focus on.

It is also recommended that the discussion be shortened to one or two paragraphs re-iterating results, then using the literature to support key findings. At this time, the first half of the discussion reads more like a results section

**We restructured the discussion to focus on the primary outcomes and the finding that water quality was not improved in intervention villages. In the first and seventh paragraph, we note where the intervention succeeded: namely, in improving WASH institutions and infrastructure. In the last paragraph, we recommend focuses for other community-led interventions.**

Reviewer #4: The paper "Effects of a community-driven water, sanitation, and hygiene intervention on diarrhea, child growth, and local institutions: a cluster-randomized controlled trial in rural

Democratic Republic of Congo" by Quattrochi et al." analyzed outcomes through a randomized controlled trial in rural areas in the Democratic Republic of Congo whether a nationally implemented WASH program affected health outcomes. The authors used the VEA program as intervention and had prolonged follow up times in order to further assess the sustainability of the program. While the background and the execution of the study is well described, the methods section could benefit from additional adjustments for clarity.

Major comments:

Methods

*	I am a bit confused about your randomization strategy. While in the Study Design you write "We stratified randomization to ensure that the intervention and control groups are balanced", the Randomization and masking section states "Clusters were randomly allocated to intervention using a random number generator" and further in the Statistical analysis you write "Sample size was based on the primary outcome of diarrhea prevalence". Please clarify your randomization strategy coherently. Furthermore, please state why you did not consider village size in the randomization of the clusters as this can have a major effect with respect to the size of the intervention and control group.

**We have redrafted the study design section to improve the clarity of our explanation, clarifying the ways in which we implemented randomization at cluster level, stratifying by both cluster size and province.**

**With respect to question of stratification by village size, we actually did something quite similar to what the reviewer has noted: While we did not directly stratify by village size, we instead stratified by number of villages per cluster (since by the study’s design, clusters could contain multiple villages). In this way we maintained similar sized clusters in both treatment and control groups.**

*	For the subgroup analysis, restricting the data to the specific subgroup and perform the analysis again is one method. However, to avoid sampling bias which might occur due to the data restriction to the subgroup, the model should be fit with a variable for each of the provinces (if province is the subgroup) and the respective coefficient should be examined. This way, sampling bias can be avoided and a more general claim can be made.

**For each subgroup analysis (by province and by child sex), we now present results from a linear model with interaction terms between the intervention and the subgroup (Tables S5 and S7). The last two paragraphs in the Results section describe these results.**

*	In your discussion you write that "changes did not result in any effect". However, this claim is not supported by your analysis. Since you use linear models, you check for the effect size to be significantly different from 0 and H0 is your coefficient estimate is 0. However, if this is the case, you can only claim that you could not find an effect vs. there is no effect because these are two different statements with different consequences. Thus, please adjust your discussion accordingly.

**We have adjusted this claim and are now careful to state only that “we cannot reject the null hypothesis that the intervention did not result in any effect on child length-for-age or diarrhea.”**

Minor comments:

General

*	While to the data is provided as [data.worldbank.org](http://data.worldbank.org/), more detail on where exactly the data can be found would be beneficial. Furthermore, a different link for getting access to the code should be provided upon publication.

**The data set and the code to replicate the findings we report in this manuscript will receive a specific URL hosted at** [**reproducibility.worldbank.org**](http://reproducibility.worldbank.org/) **upon completion of a mandatory World Bank reproducibility review, in accordance with the PLOS Medicine guidelines on data availability.**

*	For better readability, if you write numbers with decimal places, please be consistent in their writing, i.e. central point '' 2·0 billion people'' vs decimal point ''1.4 million deaths''.

**We have edited the text for consistency on this point.**

Abstract

*	Findings: include CI to signify confidence interval when reporting diarrhea prevalence.

**We have edited the text to include confidence intervals for the effect of the program on diarrhea prevalence.**

Introduction

*	Please write out the abbreviation "VEA" as this is often used later in the manuscript.

**VEA stands for “Village et Ecole Assainis” or “Healthy Villages and Schools” in English. We have replaced all instances of VEA with “Healthy Villages and Schools.”**

Methods

*	Thirty four should be written as a number 43, as only number until twelve are written as words in a flow text.

**We have made the suggested edit.**

*	The link to the implementation of the VEA program should be put as a reference and cited accordingly.

**We added a reference and cited it.**

*	Statistical analyses section: ''..we fit linear models..'' and "and fit the model described above" These sentences should be written in past tense as this is work you have done already.

**We have adjusted the text, now referring to all analysis in the past tense.**

*	You mention that your random sampling, is "likely increasing the precision of final estimates." Since you are not performing computational statistics with Monte Carlo Methods, your random sampling targets towards dividing the population into two comparable groups for assessing the intervention effects. Thus, this part of the sentence should be deleted.

**We deleted this sentence.**

*	In the statistical analysis section you state that you fitted linear models. I assume you fitted generalized linear mixed models, as the outcome variable does not always follow a normal distribution (diarrhea?) and you included random effects for the clusters. If this is the case, please state any other distributions than Gaussian for the response and change the wording to generalized linear model. Else please clarify your statistical approach.

**We clarified as follows: For both primary and secondary outcomes, whether binary or continuous, we fitted linear models with a binary variable indicating whether the participant was in a treatment or control cluster** [[1]](https://www.zotero.org/google-docs/?LYgM85)**. Because randomization was stratified by province and number-of-villages-per-cluster, we included binary variables (fixed effects) for each stratum (n=12) in the model. We also included gender and age (month) indicator variables for all child health outcomes. We clustered standard errors at the cluster (i.e. group of villages) level.**

**The citation refers to Gomila (2020), who argues that, when estimating treatment effects from an experiment with a binary outcome, linear models are preferred to logistic or probit models, because they are (i) easier to interpret, and (ii) less likely to be biased when fixed effects are used (as we did to account for how the intervention was randomly assigned).**

**Another reason that we retain our linear estimates is that we pre-registered linear models.**

*	Please state the range of the The WASH governance index. Is it a number between 0 and 100, or 1-10, or 1-50 and provide a link or a short description in the appendix how this is calculated.

**The index ranges from -2.35 to 2.12. It consists of five subcomponents. Table S1 provides summary statistics and intervention effects on each subcomponent. For each subcomponent, z-scores calculated by subtracting the control group mean from each value, and dividing by the control group standard deviation (Kling, Liebman, and Katz 2007). We normalized the index such that the mean is zero and the standard deviation is one in the control group.**

**We expanded the table notes to include details of how the index was constructed and the range.**

[**Kling JR, Liebman JB, Katz LF. Experimental Analysis of Neighborhood Effects. Econometrica. 2007;75: 83–119. doi:10.1111/j.1468-0262.2007.00733.x**](https://www.zotero.org/google-docs/?N3AMyJ)

Results

*	Table 1 would benefit from statistical tests, e.g. t-test, for differences in the groups with respect to the characteristics, as this would underpin your claim statistically.

**To Table 1 (now Table 2), we added the intervention coefficient from a regression of each variable on intervention group, with fixed effects for province-strata, and standard errors clustered at the cluster (i.e. group of villages) level (i.e. the same functional form for our ITT estimates). We also report the corresponding p-value.**

*	"mean length-for-age Z score was -2.18 (SD 1.60)", correct to "(1.60 SD)" to be consistent with your writing.

**We have corrected this in the text.**

*	For better readability state in the Methods section that the ITT (intention-to-treat) estimate corresponds to the parameter estimate of the "binary variable indicating whether the participant was in the intervention or control cluster." In this case, this number states an effect measure and is not the mean difference, as you write in the Results section. "The intervention had no effect on diarrhea (mean difference -0.01 [95% -0.05- 0.03])", in particular when diarrhea is not normal distributed. Please correct also in the abstract.

**We now refer to it as an adjusted mean difference, to reflect the fact it is a difference in means between the intervention and control groups after accounting for age and sex of child, and the stratified randomization.**

*	Correct "Villages in the intervention group had a 0.40 SD higher score" and "Intervention group respondents scored 0.23 SD" to "Villages in the intervention group had a 0.40 higher score" and "Intervention group respondents scored 0.23"

**We made these corrections.**

*	Correct "(-0.39 difference in ln(MPN); 95% CI -0.73- -0.04) ." to "(-0.39 difference in ln(MPN); 95% CI -0.73 - -0.04).".

**This sentence has been revised as we now report log10(MPN), per another reviewer’s request.**

Discussion

*	The discussion section should completely be written in past tense.

**We rewrote discussion in the past tense.**

Reviewer #5: Overall

This is an important long term follow-up of a pragmatic implementation of a government's evidence based intervention.

The conflict setting is indeed important and a big achievement for the researchers to have completed this project.

There is a major issue with studies of complex interventions and non-drug trials. There is an important move away from primary and secondary outcomes to causal pathway analysis and not seeing p-values as be all and end all.

In my view, there is too much emphasis in this complex evaluation on p-values, than on using the range of distal and proximal outcomes that exist in a causal pathway analysis that may better account for changes observed.

Having said that, the fundamental message that is striking is that the quality of water is not good, functionality of some infrastructures were not better in intervention, and knowledge did not transfer to change of behaviour. Even the infrastructures that remained, were minimal in their differences (arguably not clinically significant). All these likely to show in individual tests or a causal pathway analysis (if was done) that health outcomes are unlikely to be present.

Several questions could be better addressed in this manuscript which could allow the reader to get more out of the evidence.

Intervention

What were the average size of communities/villages as the unit which implemented for a group of people at village or neighbourhood or what level?

**The mean village size in the intervention group was 456 people (median 400; IQR 502). We added this information to the first paragraph in Procedures.**

How many families per volunteer and were the volunteers still active or the committees still active - it seems these were assessed but reporting was not clear on the outcome of these

**Mean village size of 456 and mean household size of 7 implies 65 families per village. In each village, approximately 20 volunteers were trained (Table 1).**

What were the infrastrucures and what funds were available for these after the first year? It seems the water points n the control communities are just as many - this will cause no difference between arms for the outcomes measured but there is little discussion of this.

**There were no funds available after the intervention was completed, which took an average of 6 months in our sample.**

**According to the program, the NGO in charge of infrastructure construction was assigned the mission of repairing free of charge all water points that may have been broken down within one year following the end of the intervention.**

**Households in the intervention group were 24 percentage points more likely to report using an improved water source, compared to the control group. However, the time spent collecting water was statistically indistinguishable between the two groups.**

What follow-up by the government or NGOs or anyone made for the sake of these projects in 3 years irrespective of water points, in terms of quality of water, behaviour change and community monitoring of each other etc ? (Any and best activities or training, if left without follow-up and encouragement, tend to become less effective, even a well trained doctor without input from colleagues and equipment and functioning clinic will wither and become ineffective) It is not clear what has been done in 3 years to support these communities - if nothing, then the findings are not surprising - this is one of the tragedies of development for many such projects.

**Health zones and health areas (sub-divisions of health zones) are the government entities closest to communities. Health zones are led by a Chief Medical Officer (MCZ) and supported by a Water and Sanitation Supervisor (SEA) and a Community Animator (AC). Health areas, operating under the health zones, are managed by a Head Nurse and an Assistant Nurse. We asked each village if any of these agents had visited, and, if so, in what year.**

**From 2020 to 2022, the Chief Medical Officer visited 18% of the villages, the Water and Sanitation Supervisor visited 32%, and 44.4% of communities were visited by the Community Animator. Additionally, 61% of the communities received visits from the Head Nurse.**

**Under the Healthy Villages & Schools program, implementing NGOs were required to revisit villages for free repairs, corrections, construction, or support within a year after the program's implementation. Our survey, conducted 3.5 years after the program, documented that 18 villages had been revisited by the NGOs. Of these, 7 visits were for sensitization activities, 12 were for repairs to water points, and 2 involved the construction of new water points.**

**Between late 2019 and 2022, 38 out of 144 villages (26.4%) were revisited as part of post-certification activities. This system, a component of the Healthy Villages & Schools program, helps villages assess their adherence to health norms. In principle, these visits should occur every six months, but due to budget constraints and logistical challenges they occur much less often. These evaluations are conducted by health zone and health area officials, with results shared publicly. If any norms are not maintained, a recovery plan is proposed.**

Assessment

1. what type of observation was actually conducted that did not have reactivity biases - did the consent or training of field-assessors allow for masking of assessment intentions for the households.

**We agree with the reviewer that biases of this nature are important and we assume that all self-reported data may suffer from reactivity bias. We address this as follows: we added structured observations of handwashing to our data collection efforts; structured observations may also suffer from reactivity bias, but research has shown has less reactivity bias than self-reports (Biran et al 2008; Cousens et al 1996). Additionally, one primary outcome is not self-reported (child length-for-age), and therefore is unlikely to suffer from reactivity bias. Furthermore, several secondary outcomes are not self-reported: type of water source; type of sanitation facility; water quality; child weight-for-age; child weight-for-length. Finally, reactivity biases can affect both treatment and control. By randomly assigning the intervention, we reduce the likelihood that reactivity bias invalidates our findings.**

**Biran A, Rabie T, Schmidt W, Juvekar S, Hirve S, Curtis V. Comparing the performance of indicators of hand-washing practices in rural Indian households. Trop Med Int Health. 2008;13:278–285. doi: 10.1111/j.1365-3156.2007.02001.x.**

**Cousens S, Kanki B, Toure S, Diallo I, Curtis V. Reactivity and repeatability of hygiene behaviour: structured observations from Burkina Faso. Soc Sci Med. 1996;43:1299–1308. doi: 10.1016/0277-9536(95)00380-0.**

2. Were the field-assessors blind to the intervention or not?

**No. We state in Randomization and Masking that “Due to the participatory and visible nature of the intervention, neither participants nor data collectors were masked to treatment status.”**

Outcomes

Diarrhoea was very high - it is not possible that 40% of children had diarrhoea in a week even in a conflict zone at high diarrhoea season.

**We agree that a priori this seems like a very high prevalence. However we note that in the 2013-14 DHS (most recent available for DRC), 28.2% of children aged 12-23 months and 33% of children aged 6-11 months had diarrhea in the previous two weeks. Therefore, we find it plausible that in the more-disadvantaged rural areas that were eligible for Healthy Villages & Schools (they were eligible in part due to serious challenges with WASH services) the prevalence of diarrhea in the last seven days could reach 40%.**

It is very well established that reported diarrhoeas are very poor measures of diarrhoea and especially if definition is not well explained - these figures are very odd, even if the overall results of no difference between arms is understandable.

**We agree that accurate measurement of diarrhea can be challenging and for this reason we took care to follow best practice for elicitation of caregiver reports of diarrhea. We followed the following procedure; The female head of household was asked about all children living in the household. She was interviewed once, in her home. She was asked to respond yes or no to a list of symptoms in the last seven days.
We coded children as having had diarrhea if the respondent said:
1. Yes to ‘diarrhea’ , or
2. Yes to ‘three or more stools’ AND ‘watery or soft stool’, or**

**3. Yes to blood in the stool**

Discussion and conclusion -

Although it is clear that these settings are very complex and other risks affect outcomes such as open defecation on going to the field for farming, there are many other things that affect outcomes and make the situation complex - these include open defaecation on travelling between villages, or food safety/hygiene, children playing on dirt and geophagy etc all have additional risks, but it is important to note that this is an RCT and so these factors should cancel out. There is little discussion on any contamination to other villages in the control arm and if this was not possible due to infrastructure resources, then what about the behaviour related issues. For example it seems spending was same between 2 arms too as was time taken to fetch water and availability of water points.

**We agree that contamination/spillover is certainly a potential issue in this kind of WASH intervention. For this reason, we avoided having treatment villages located within close proximity to control villages, which would have made this kind of experimental interference more likely. For example, as described in more detail in Randomization and Masking, we conducted a clustered randomization which did not allow any control villages to be within 2.5 kilometers of treatment villages, because if they were within this proximity they would be more likely to benefit from new water infrastructure in treatment villages, thereby attenuating any treatment effect.**

Any attachments provided with reviews can be seen via the following link:

--------------------------------------------------------- ---

General editorial requests:

(Note: not all will apply to your paper, but please check each item carefully)

*	One of our most important requests is to do with the study registration. We thank the authors for their openness and transparency with this. We kindly ask that, in the Methods section of your revised manuscript, you describe the particulars surrounding the study registration for both studies (including the timing of registration in relation to patient recruitment and explain the specific reasons for failing to register the work prior to patient enrolment). We also ask that you write a sentence to confirm that all studies are now registered and that any future work will be registered prospectively.

**We revised the Study Design section to better explain study registration, and we added a sentence confirming that all studies are now registered and that any future work will be registered prospectively.**

*	We ask every co-author listed on the manuscript to fill in a contributing author statement, making sure to declare all competing interests. If any of the co-authors have not filled in the statement, we will remind them to do so when the paper is revised. If all statements are not completed in a timely fashion this could hold up the re-review process. If new competing interests are declared later in the revision process, this may also hold up the submission. Should there be a problem getting one of your co-authors to fill in a statement we will be in contact. Please do not add or remove authors without first discussing this with the handling editor. You can see our competing interests policy here: <http://journals.plos.org/plosmedicine/s/competing-interests>.

*	Please upload any figures associated with your paper as individual TIF or EPS files with 300dpi resolution at resubmission; please read our figure guidelines for more information on our requirements: <http://journals.plos.org/plosmedicine/s/figures>. While revising your submission, please upload your figure files to the PACE digital diagnostic tool, <https://pacev2.apexcovantage.com/>. PACE helps ensure that figures meet PLOS requirements. To use PACE, you must first register as a user. Then, login and navigate to the UPLOAD tab, where you will find detailed instructions on how to use the tool. If you encounter any issues or have any questions when using PACE, please email us at PLOSMedicine@plos.org.

*	Thank you for completing a CONSORT checklist. Could you kindly revise the checklist, using section and paragraph numbers, rather than page numbers, please? (Page numbers are likely to change later down the production process.) Please also add a statement to the Methods that highlights that you have completed such a checklist, e.g. "This study is reported as per CONSORT guideline (S1 Checklist)”, or similar.

**We have included the suggested statement in the Methods section.**

FORMATTING - GENERAL

*	Abstract: Please structure your abstract using the PLOS Medicine headings (Background, Methods and Findings, Conclusions). Please combine the Methods and Findings sections into one section.

**We have restructured the abstract to follow the PLOS Medicine headings.**

*	At this stage, we ask that you include a short, non-technical Author Summary of your research to make findings accessible to a wide audience that includes both scientists and non-scientists. The Author Summary should immediately follow the Abstract in your revised manuscript. This text is subject to editorial change and should be distinct from the scientific abstract. Ideally each sub-heading should contain 2-3 single sentence, concise bullet points containing the most salient points from your study. In the final bullet point of 'What Do These Findings Mean?', please include the main limitations of the study in non-technical language. Please see our author guidelines for more information: <https://journals.plos.org/plosmedicine/s/revising-your-manuscript#loc-author-summary>.

**We have included the Author Summary after the abstract.**

*	Please express the main results with 95% CIs as well as p values. When reporting p values please report as p<0.001 and where higher as the exact p value p=0.002, for example. Throughout, suggest reporting statistical information as follows to improve clarity for the reader "22% (95% CI [13%,28%]; p</=)". Please be sure to define all numerical values at first use.

*	Please include page numbers and line numbers in the manuscript file. Use continuous line numbers (do not restart the numbering on each page).

**We have added page numbers and line numbers.**

*	Please cite the reference numbers in square brackets. Citations should precede punctuation.
**We now use square brackets and citations precede punctuation.**

FIGURES AND TABLES

*	Please provide titles and legends for all figures and tables (including those in Supporting Information files).

*	Please define all abbreviations used in each figure/table (including those in Supporting Information files).

*	Please consider avoiding the use of red and green in order to make your figure more accessible to those with color blindness.

SUPPLEMENTARY MATERIAL

*	Please note that supplementary material will be posted as supplied by the authors. Therefore, please amend it according to the relevant comments outlined here.

*	Please cite your Supporting Information as outlined here: <https://journals.plos.org/plosmedicine/s/supporting-information>

REFERENCES

*	PLOS uses the numbered citation (citation-sequence) method and first six authors, et al.

*	Please ensure that journal name abbreviations match those found in the National Center for Biotechnology Information (NCBI) databases (<http://www.ncbi.nlm.nih.gov/nlmcatalog/journals>), and are appropriately formatted and capitalised.

*	Where website addresses are cited, please include the complete URL and specify the date of access (e.g. [accessed: 12/06/2023]).

*	Please also see <https://journals.plos.org/plosmedicine/s/submission-guidelines#loc-references> for further details on reference formatting.

RCTs

*	PLOS Medicine requires that all trials be prospectively registered in one of registries recognized by WHO. Please ensure that study registration details are included in the Methods section.

**We have significantly expanded our description of our study registrations in the Study Design section of the manuscript.**

*	Please structure the Methods section using the following sub-headings: Study design and participants, Randomization and masking, Procedures, Outcomes, Statistical analysis.

**We have re-structured the Methods section following the suggested sub-headings.**

*	Please specify the dates (Month Day, Year) during which study enrollment and follow up occurred.

**This information is included at the beginning of the Results section.**

*	Please include absolute numbers wherever you report percentages; eg, n/N (%)

**We report N and %.**

*	Please present the safety data for the study including numbers of specific events and whether or not adverse events are thought to be related to treatment. AEs should be reported in the abstract, per CONSORT and CONSORT-Harms.

*	If your trial had to undergo important modifications in response to extenuating circumstances, please complete the CONSERVE-CONSORT checklist and provide in your Supporting Information; (<https://www.equator-network.org/reporting-guidelines/guidelines-for-reporting-trial-protocols-and-completed-trials-modified-due-to-the-covid-19-pandemic-and-other-extenuating-circumstances-the-conserve-2021-statement/>). When completing the checklist, please use section and paragraph numbers, rather than page numbers.

**No modifications due to extenuating circumstances took place.**

*	In keeping with our commitment to Open Science, please include the study protocol document and analysis plan (including any amendments) as Supporting Information to be published with the manuscript if accepted.

**We uploaded (1) our study protocol and analysis plan from 2020, (2) our study protocol and analysis plan from 2023, (3) our concept note from 2019**
