# Supplementary material for: Effects of a community-driven water, sanitation, and hygiene intervention on diarrhea, child growth, and local institutions: A cluster-randomized controlled trial in rural Democratic Republic of Congo
Source: PLoS Med. 2025 Mar 6;22(3):e1004524. doi: 10.1371/journal.pmed.1004524 (PMC11884671; doi:10.1371/journal.pmed.1004524)
Supplement: Attachment — Submitted filename: PLOS Medicine 2nd round response memo.docx [file pmed.1004524.s013.docx]

Response to second round of editorial and reviewer comments
PMEDICINE-D-24-02389R2 Decision - [EMID:073cf9264f816bb0]
Effects of a community-driven water, sanitation, and hygiene intervention on diarrhea, child growth, and local institutions: a cluster-randomized controlled trial in rural Democratic Republic of Congo

**We thank the editors and reviewers for another round of helpful comments and suggestions. We addressed them below, in bold.**

------------------------------------------------------------

Requests from Editors:

GENERAL EDITORIAL REQEUSTS

* Please confirm that your title complies with to PLOS Medicine's style. Your title must be nondeclarative and not a question. It should begin with main concept if possible. "Effect of" should be used only if causality can be inferred, i.e., for an RCT. Please place the study design ("A randomized controlled trial," "A retrospective study," "A modelling study," etc.) in the subtitle (ie, after a colon).

**Our title complies.**

* Please confirm that your abstract to complies with our requirements, including providing all the information relevant to this study type <https://journals.plos.org/plosmedicine/s/submission-guidelines#loc-abstract>

**We edited the abstract to include all relevant information.**

* Please ensure that the Introduction ends with a clear description of the study question or hypothesis.

**The last sentence of the Introduction reads: Our goal was to estimate the effect of Healthy Villages & Schools on diarrhea prevalence, child length-for-age, and WASH institutions.**

* Please ensure that all abbreviations are defined at first use throughout the text.

**We have defined all abbreviations.**

FUNDING STATEMENT

* The funding statement should include: specific grant numbers, initials of authors who received each award, URLs to sponsors’ websites. Also, please state whether any sponsors or funders (other than the named authors) played any role in study design, data collection and analysis, the decision to publish, or preparation of the manuscript. If they had no role in the research, include this sentence: “The funders had no role in study design, data collection and analysis, decision to publish, or preparation of the manuscript.”

**We revised our statement to*:*
This study was funded by the UK Foreign, Commonwealth, and Development Office (FCDO) (**[**https://www.gov.uk/government/organisations/foreign-commonwealth-development-office**](https://www.gov.uk/government/organisations/foreign-commonwealth-development-office)**), via Amendment No. 3 to the Supplemental Arrangement with the World Bank regarding Multi-Donor Trust Fund for Impact Evaluation to Development Impact (TF072617, parallel to TF072161). AC, KC, and EM were staff at Development Impact at that time. The Healthy Villages & Schools program was a DRC Government national program funded by UK’s FCDO and implemented with UNICEF’s support. The funder and implementing partners provided inputs at the design stage to ensure the study addressed policy and program priorities of importance to them. The funders had no role in data collection and analysis, decision to publish, or preparation of the manuscript.**

COMPETING INTERESTS STATEMENT

* All authors must declare their relevant competing interests per the PLOS policy, which can be seen here: <https://journals.plos.org/plosmedicine/s/competing-interests> For authors with ties to industry, please indicate whether any of the interests has a financial stake in the results of the current study.

**All authors have declared.**

DATA AVAILABILITY

* Thank you for agreeing to make your data available. At this time, please provide the link to the data repository and accession numbers required for access.

**The data repository is here:** [**https://doi.org/10.60572/q3tb-tw37**](https://doi.org/10.60572/q3tb-tw37)

**Currently, if you click on any of the datasets, it states: “Data is forthcoming in the World Bank Microdata Library. Users can contact Aidan Coville (acoville@worldbank.org) for earlier access.”**

**We anticipate that the data will be uploaded and available without restrictions by late January 2025. By that time, the code and data will have been independently assessed by a World Bank team to ensure computational reproducibility, according to the protocols outlined here:** [**https://worldbank.github.io/wb-reproducible-research-repository/**](https://worldbank.github.io/wb-reproducible-research-repository/)**"**

FIGURES

* Where data points are discrete, please ensure that they are depicted in the figures as discrete data and not as a continuous line.

**Our only figure consists of two kernel density distributions of length-for-age z-scores. To our knowledge, there is no way to represent the information in this figure with discrete data points. Density distributions are the most transparent way to illustrate how data are spread.**

Comments from Reviewers:

Reviewer #1: The authors have sufficiently responded to my comments, though the resubmission file did not include the Tables so I was unable to review changes to those. Overall, I still don't feel the manuscript is sufficiently novel to warrant publication in PLOS Medicine given other similar research in this area.

**We thank Reviewer #1 for their comments.**

Reviewer #2: General comments

The authors have done a very good job to improve the reporting of this cluster randomized trial. Despite a slightly messy pre-registration and randomization process (at least by biomedical clinical trial standards, which are relevant here), the overall inference from the trial should be reasonable, and the transparent reporting in the revised paper helps clarify what was done and why some of the design characteristics are slightly unintuitive. The authors should be commended on fielding a trial under such challenging circumstances, and for doing a very nice, comprehensive job to respond to referee comments on the previous draft. I had just two small comments on the revised draft. I hope these comments are helpful

Ben Arnold, UCSF

MAJOR comments

(1) Line 195

"In Kasai Central, due to budget constraints, we increased the probability of being selected into the control group proportionally to reflect the fact that only 16 out of 81 villages could receive the intervention."

Please specify the allocation probabilities for the intervention and control groups in Kasai Central.

**We changed the relevant sentences to: “In Kasai Central, due to budget constraints, we increased the probability of being selected into the control group to 75%, to reflect the fact that only 16 out of 81 villages could receive the intervention. Thus the allocation probability for the intervention group was 25%.”**

MINOR comments

(2) I may have missed it in the SI, but in the results it would be helpful for the authors to report the estimated ICC for diarrhea and LAZ, their primary health outcomes. With much higher precision than planned for in the design, it seems like the ICC for diarrhea might have been lower than the one they assumed in the design (ICC=0.09), and reporting such information is helpful for planning of future studies. From Murray 2004 (an old paper, but important paper <https://pmc.ncbi.nlm.nih.gov/articles/PMC1448268/>): "Because the ICC of concern in any GRT is the ICC as it operates in the primary analysis, these findings reinforce the need for investigators to use estimates in their power analyses that closely reflect the endpoints, target population, and primary analysis planned for the trial. And while the sources just cited will help considerably in this regard, we join others who have urged publication of such estimates as a routine part of reporting the results of GRTs."

**We added ICCs to Table 3, where we report treatment effects on our primary outcomes.**

**We thank Reviewer #2 for their comments.**

Reviewer #4: The authors have provided detailed responses to the questions and comments and have incorporated them thoroughly into their manuscript. In my opinion, there are no further objections that would prevent publication.

**We thank Reviewer #4 for their comments.**
